# Fluorescence/photoacoustic imaging-guided nanomaterials for highly efficient cancer theragnostic agent

**DOI:** 10.1038/s41598-021-95660-w

**Published:** 2021-08-05

**Authors:** Vu Hoang Minh Doan, Van Tu Nguyen, Sudip Mondal, Thi Mai Thien Vo, Cao Duong Ly, Dinh Dat Vu, Gebremedhin Yonatan Ataklti, Sumin Park, Jaeyeop Choi, Junghwan Oh

**Affiliations:** 1 Industry 4.0 Convergence Bionics Engineering, Department of Biomedical Engineering, Pukyong National University, Busan, 48513 Republic of Korea; 2 New-Senior Healthcare Innovation Center (BK21 Plus), Pukyong National University, Busan, 48513 Republic of Korea; 3 Ohlabs Corp., Busan, 48513 Republic of Korea

**Keywords:** Imaging techniques, Chemical modification, Breast cancer

## Abstract

Imaging modalities combined with a multimodal nanocomposite contrast agent hold great potential for significant contributions in the biomedical field. Among modern imaging techniques, photoacoustic (PA) and fluorescence (FL) imaging gained much attention due to their non-invasive feature and the mutually supportive characteristic in terms of spatial resolution, penetration depth, imaging sensitivity, and speed. In this present study, we synthesized IR783 conjugated chitosan–polypyrrole nanocomposites (IR-CS–PPy NCs) as a theragnostic agent used for FL/PA dual-modal imaging. A customized FL and photoacoustic imaging system was constructed to perform required imaging experiments and create high-contrast images. The proposed nanocomposites were confirmed to have great biosafety, essentially a near-infrared (NIR) absorbance property with enhanced photostability. The in vitro photothermal results indicate the high-efficiency MDA-MB-231 breast cancer cell ablation ability of IR-CS–PPy NCs under 808 nm NIR laser irradiation. The in vivo PTT study revealed the complete destruction of the tumor tissues with IR-CS–PPy NCs without further recurrence. The in vitro and in vivo results suggest that the demonstrated nanocomposites, together with the proposed imaging systems could be an effective theragnostic agent for imaging-guided cancer treatment.

## Introduction

Recently, breast cancer is the main cause of mortality among women worldwide^1^. The dramatic rise in breast cancer death rates over the last two decades necessitates a greater emphasis on finding an adaptive therapy^2^. The primary target in the battle against breast cancer is to establish successful therapeutic strategies with low toxicity and high precision for eradicating tumors, especially their metastases, and preventing recurrence^3^. However, conventional methods for treating cancer such as chemotherapy, radiotherapy have several side effects^4,5^ and may need surgical intervention required for cancer treatment^6,7^. Photothermal therapy (PTT), which produces heat from photothermal agents upon near-infrared (NIR) irradiation, is a promising technique of cancer management^8^. PTT has many benefits over conventional cancer treatments, including high tumor sensitivity; temporal, spatial selectivity; and less invasive to normal surrounding tissues^9^. The use of PTT agents to ablate tumors by achieving adequate hyperthermia using NIR laser irradiation has been investigated as a highly accurate and minimally invasive cancer treatment procedure^10–12^.

Photothermal therapy agents have a high NIR absorption property which facilitates deeper penetration into the solid tumor without dispersing absorbed photons to the surrounding normal tissues^13,14^. Several NIR-absorbing materials have been investigated for photothermal applications, including gold nanorods^15,16^, upconversion nanoparticles^17^, carbon nanotubes^18^, graphene^19^, graphene–iron oxide nanoparticles^20^, and polypyrrole nanoparticles (PPy NPs)^21,22^. PPy NPs are one of the most effective polymeric photothermal agents among those materials. They have high NIR absorbance, effective photothermal conversion efficiency, biocompatibility, and low cytotoxicity. Additionally, PPy NCs show excellent photostability when exposed to NIR for a long time period^23^. PPy NPs are easy to fabricate with large quantities at affordable price^24^. Furthermore, PPy NPs were used for cancer diagnosis as effective contrast agents because of their high NIR absorption properties^25,26^. A variety of research groups have used them in imaging-guided PTT for in vivo cancer treatment^27,28^. Similarly, marine biopolymers such as chitosan (CS) have attracted considerable attention in recent years for their application in the cosmetics, nutraceutical, and pharmaceutical industries^29,30^. Manivasagan et al. reported the combination of CS and PPy to be a strong NIR absorbance PTT agent for cancer treatment. The chitosan–polypyrrole nanocomposites (CS–PPy NCs) have promising efficiency which could act as a multifunction theragnostic agent for biomedical applications.

Fluorescence imaging along with NIR probes is evolving as a powerful diagnosis method for clinical applications such as intraoperative tumor margin detection^31,32^. In clinical trials, several research groups have demonstrated effective NIR fluorescence imaging to classify neoplastic tissues based on various NIR probes^33–35^. However, owing to strong optical scattering in biological tissue, fluorescence imaging resolution decreases drastically as sample depth increases^36^, restricting the efficient imaging-depth capacity. Photoacoustic imaging is a special non-invasive imaging technology that relies on the acoustic waves produced from the absorption of pulsed laser energy by endogenous tissue chromophores or supportive contrast agents^37,38^. In addition to possessing high spatial resolution, high sensitivity properties as a general optical imaging technique, photoacoustic imaging has the foremost benefit of penetration depth (up to several centimeters) since the emitted ultrasound waves are less scattered in biological tissue than optical waves^39,40^. By contrast, fluorescence imaging is beneficial for constructing large field-of-view images. Therefore, in terms of imaging resolution, depth, sensitivity, and speed, these two imaging modalities are complementary^41^. As a consequence, design and synthesis of photoacoustic/fluorescence dual-modality nanoprobes are highly desirable for many molecular imaging applications.

In this present study, the PA and FL imaging systems were fabricated to diagnose the cancer cells and tumor region treated with synthesized IR-CS–PPy NCs. The IR-CS–PPy NCs were synthesized by the following previous protocol with further modification. The PTT-assisted IR-CS–PPy NCs were verified in vitro and in vivo to confirm the photothermal effect under NIR laser irradiation. The FL/PA experiments were also performed to prove the multimodal imaging ability of the developed IR-CS–PPy NCs.

**Figure 1:**
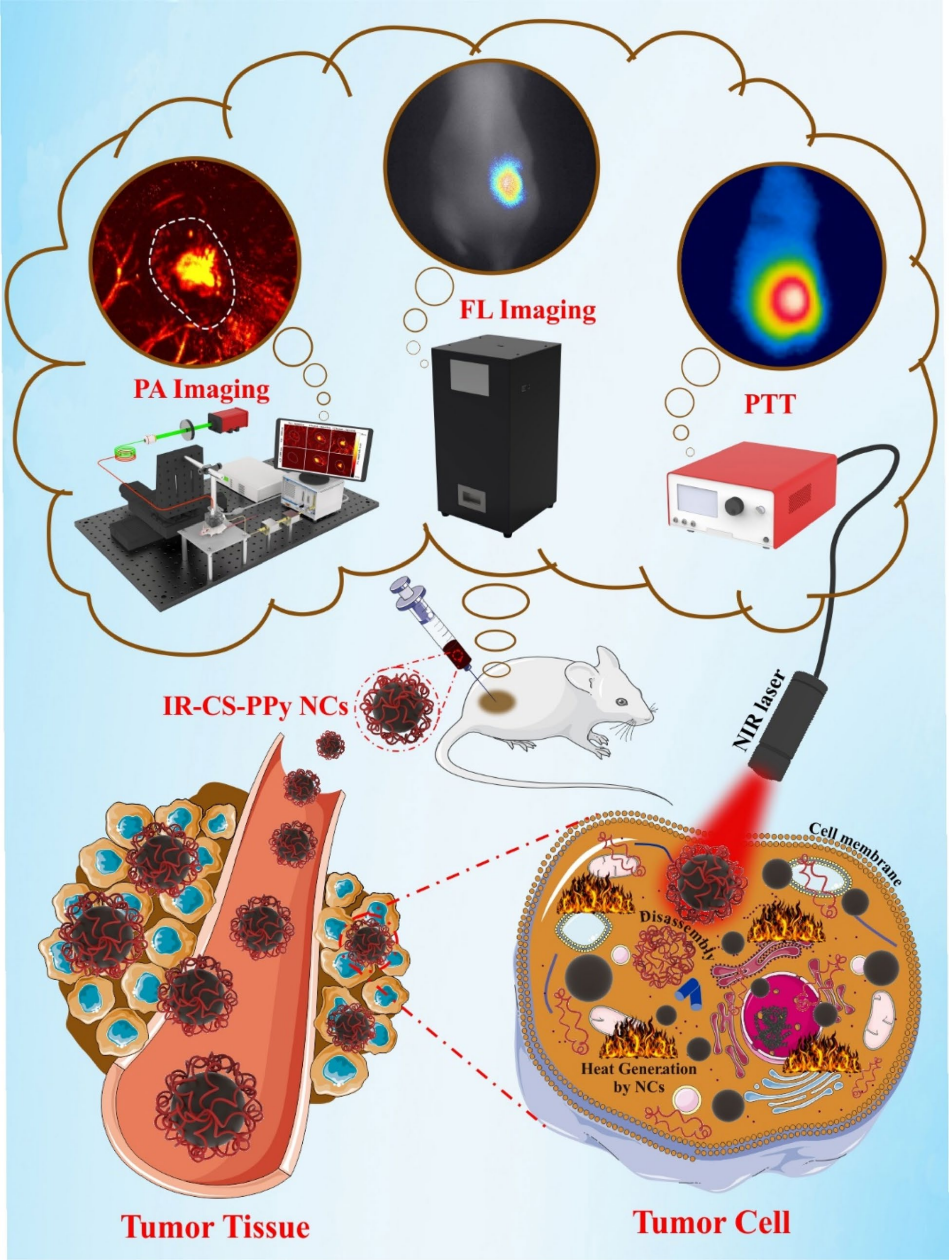
Schematic representation of IR-CS–PPy NCs for dual-channel fluorescence/photoacoustic imaging-guided photothermal therapy. The scheme was created using Servier Medical Art (http://smart.servier.com/).

Figure 1 indicates the possible mechanism of IR-CS–PPy NCs for treating the tumor with the guidance of fluorescence and photoacoustic imaging system. The IR-CS–PPy NCs characteristics required for PTT and imaging stages were described below.

## Results and discussion

### Characterization of CS–PPy NCs and IR-CS–PPy NCs

**Figure 2:**
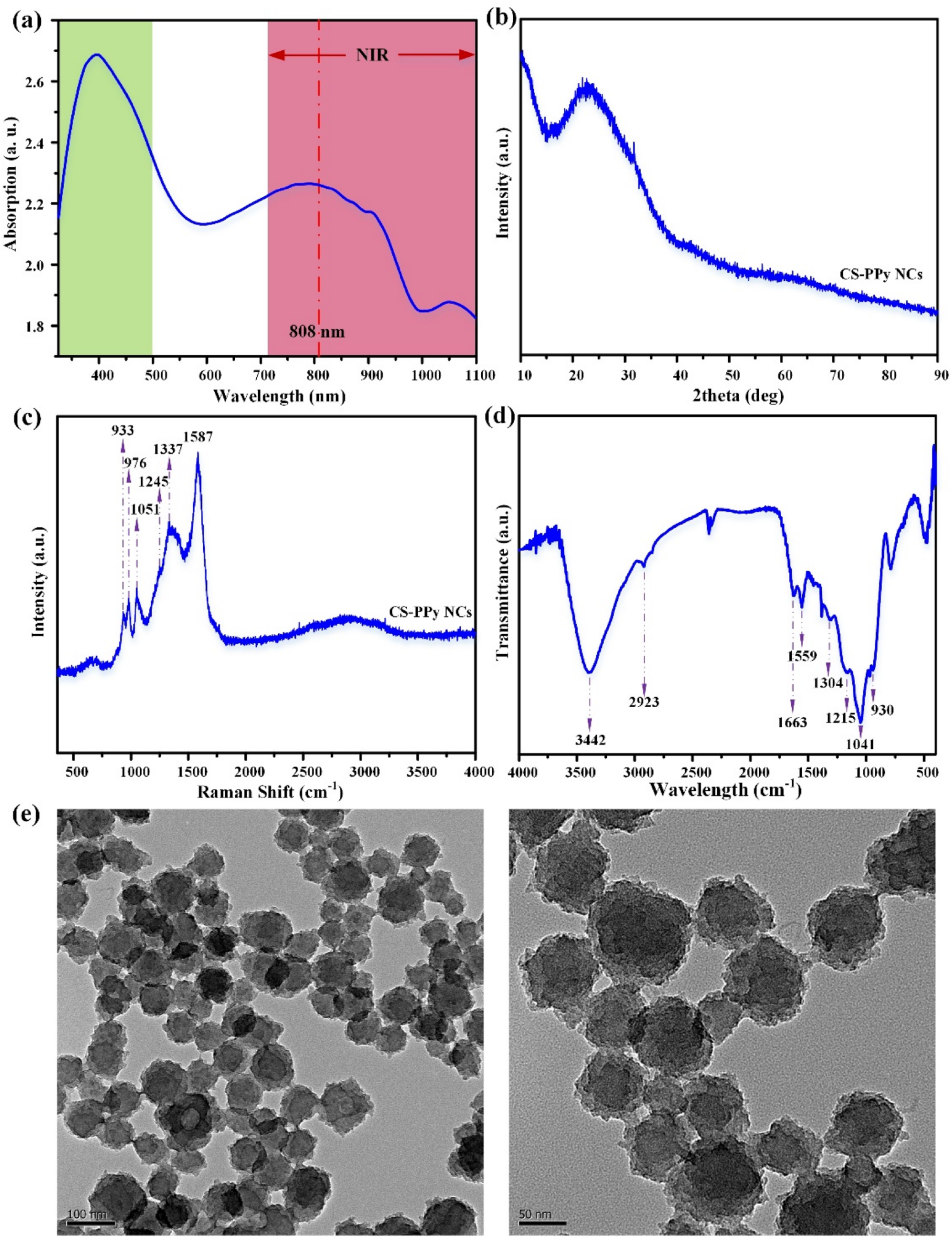
Characterization of CS–PPy NCs (**a**) UV–Vis–NIR absorbance spectrum CS–PPy NCs dispersion in water. (**b**) XRD patterns of CS–PPy NCs. (**c**) Raman spectrum of CS–PPy NCs. (**d**) FTIR spectrum of CS–PPy NCs. (**e**) FE-TEM image of CS–PPy NCs.

The UV–Vis spectra of chitosan polypyrrole nanoparticles was measured from 320 to 1100 nm wavelength (Fig. 2a). The composite CS–PPy NCs indicate a significant broad absorption band at 320–500 nm. The absorption band recorded at 320 nm indicates the π–π* transition of the heteroatom pyrrole ring^42^. The samples exhibit weak polaron and bipolaron absorption bands at ~ 620 nm to ~ 900 nm, which results in broadband absorption^43^. The band intensity is directly proportional to the presence of the charge carriers due to chitosan molecules in the polymer^44,45^. The XRD analysis of CS–PPy NCs revealed an amorphous state, which could be easily identified as there are no sharp peaks recorded in the diffraction pattern (Fig. 2b). A broad peak at approximately 20°–30° (2 theta angle) is observed, which correlates with amorphous polypyrrole polymer. There are no significant additional peaks observed due to the presence of chitosan, which is also amorphous in nature. The morphological study of the CS–PPy NCs was performed through FE-TEM analysis. The CS–PPy NCs appear to be near-spherical in shape, with a size distribution of 86 ± 6 nm (Fig. 2e).

The Raman study of the synthesized CS–PPy NCs was performed to reveal the structural identity (Fig. 2c). The Raman study reveals the characteristics peaks at 1587 cm^−1^ is assigned to the C=C stretching vibration. The peak at 1337 cm^−1^ is assigned to C–C stretching vibration^46^. C–N stretching vibration mode was identified at 1051 cm^−1^ peak position of the FTIR study. The peak at 1245 cm^−1^ represents the C–H in-plane bending vibration. The peak at 933 cm^−1^ is assigned to C–H ring deformation vibration^47^. The FTIR analysis of the synthesized CS–PPy NCs was performed to reveal the functional group's identity (Fig. 2d). The peak was assigned at 3442 cm^−1^ due to the O–H vibrational mode. The characteristic peaks of pyrrole rings were recorded at 2923, 1559, 1304, 1215, 1178, 1041, and 930 cm^−1^, respectively. The peaks at 1041 cm^−1^ and 1215 cm^−1^ correspond to the C–N stretching of aliphatic amines present in the chitosan. The bands at 967 and 798 cm^−1^ are associated with out-of-plane C–H bending^48^. The N–O asymmetric stretching band was recorded at 1556 cm^−1^ assigned to the nitro compounds. As a result of the atmospheric CO_2_ interference, a small peak at 1715 cm^−1^ (C=O) was observed. Peak recorded at 2258 cm^−1^ reveals the C≡C stretching of alkyne groups, whereas, C–H stretching of alkanes was detected at a 2990 cm^−1^ wavelength.

**Figure 3:**
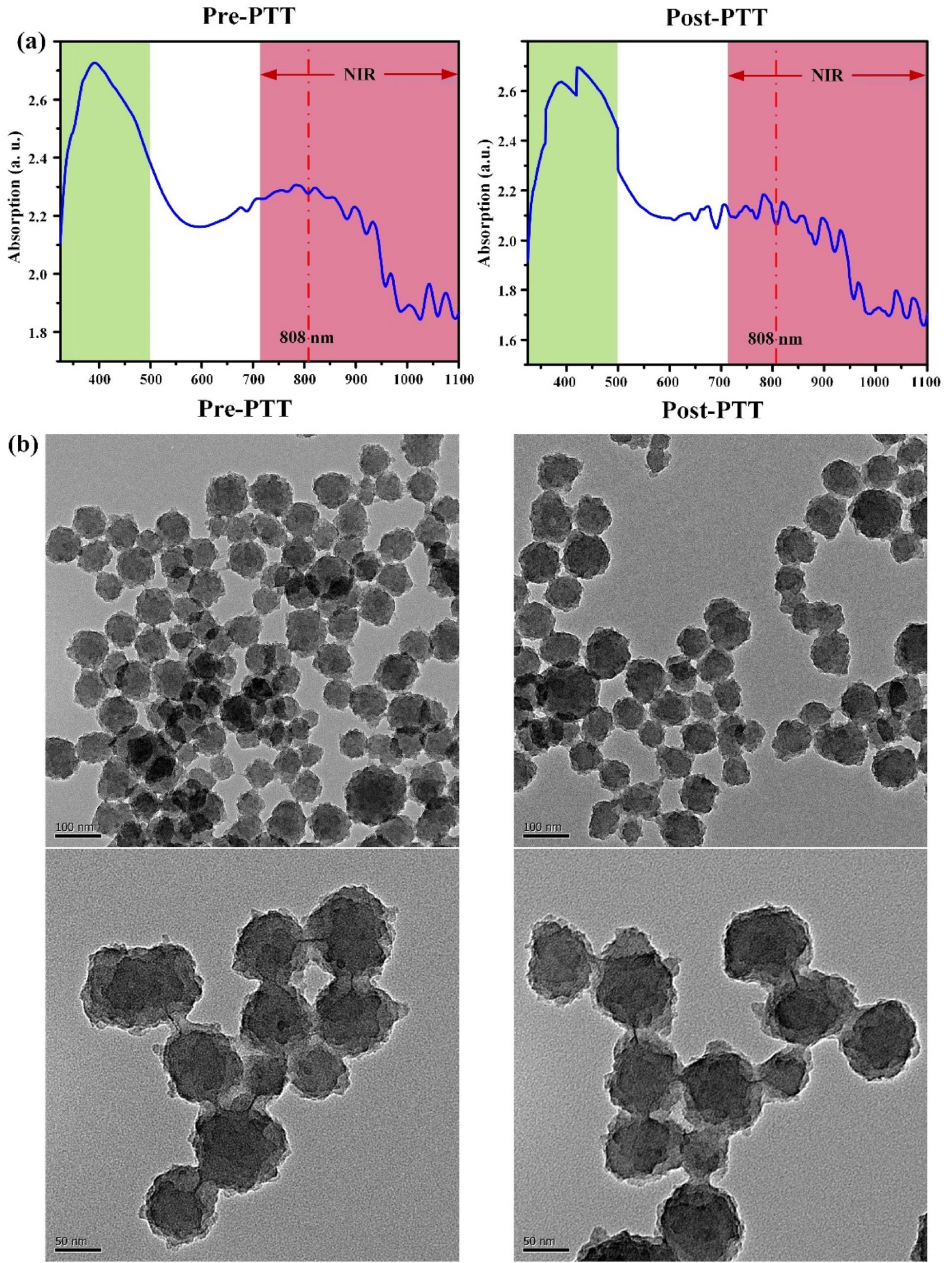
Characterization of IR-CS–PPy NCs (**a**) UV–Vis spectrum of IR-CS–PPy NCs (125 μg/mL) before and after PTT. (**b**) FE-TEM images of IR-CS–PPy NCs (125 μg/mL) before and after PTT.

The UV–Vis spectra of IR783 conjugated chitosan polypyrrole nanocomposites (IR-CS–PPy NCs) were further studied from 320 to 1100 nm wavelength (Fig. 3a). Comparing with only CS–PPy NCs, the absorption peak for IR783 was identified range of 700 nm to 900 nm wavelength. There are no absorption changes in IR-CS–PPy NCs in the region of 320 to 500 nm wavelength. The study further extended with the characterization of post-PTT IR-CS–PPy NCs by UV–Vis spectra. The post-PTT study revealed a little difference in the IR-CS–PPy NCs peak, which may result due to the high temperature mediated degradation of CS nanoparticles during PTT^49^.

The FE-TEM study revealed that there are no significant changes in morphologies of the synthesized CS–PPy NCs and IR-CS–PPy NCs. The size distributions for both nanocomposites are almost similar (86 ± 6 nm). The study extended with FE-TEM analysis for pre and post-photothermal treated IR-CS–PPy NCs. Interestingly, there are no differences observed between these two types of nanoparticles after treating with the 808-nm laser (Fig. 3b). Due to the morphological stability, the synthesized nanoparticles give good photothermal efficiency for effectively treating cancer by thermal ablation.

### Photothermal performance of IR-CS–PPy NCs

**Figure 4:**
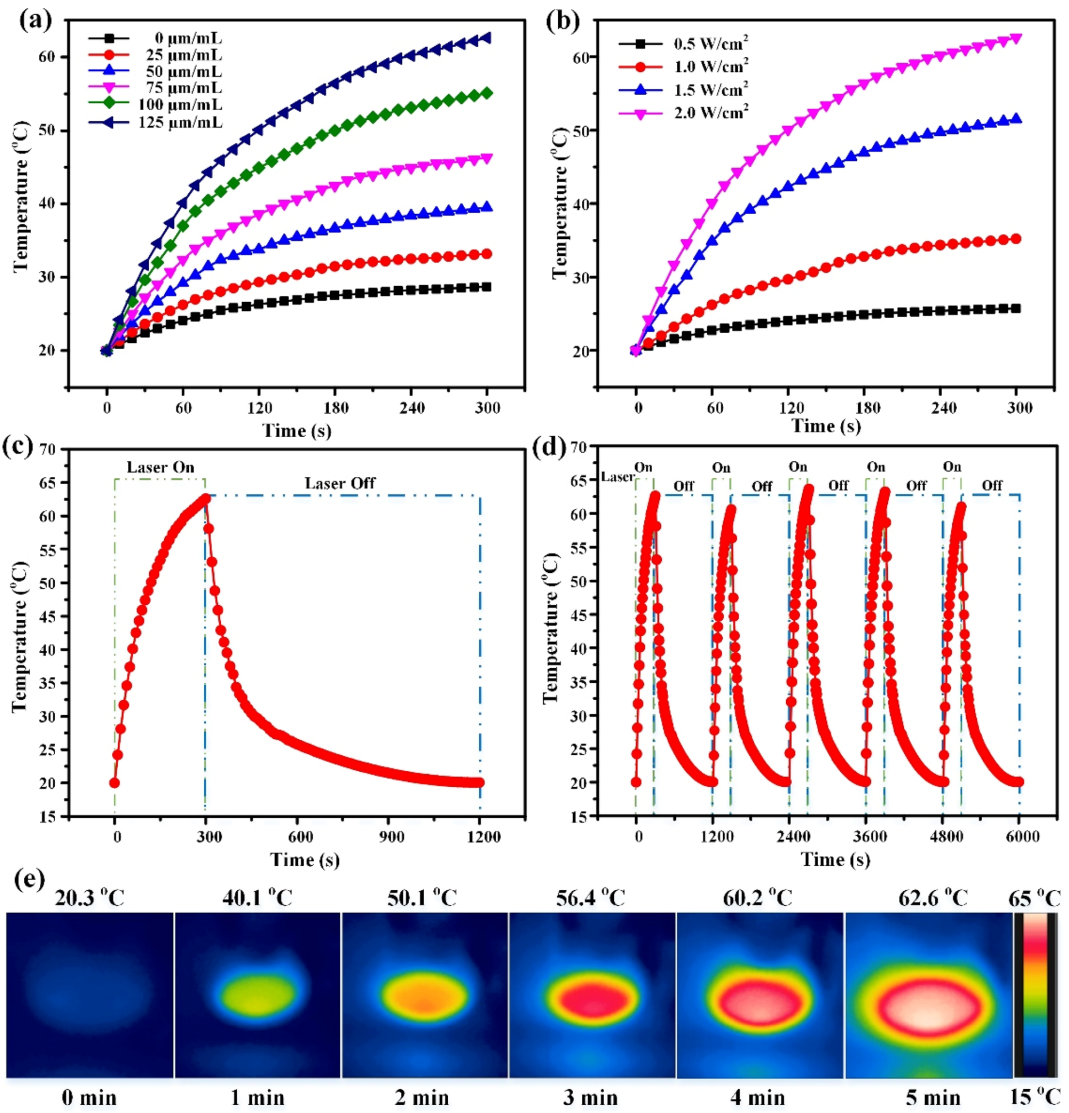
(**a**) The temperature elevation in an aqueous solution of different IR-CS–PPy NCs concentrations (0, 25, 50, 75, 100, 125 μg/mL) as a function of irradiation time by 808 nm laser at 2 W/cm^2^ of laser power densities. (**b**) The temperature elevation of IR-CS–PPy NCs aqueous solution at a concentration of 125 μg/mL under 808-nm laser irradiation at different power densities (0.5 W/cm^2^, 1.0 W/cm^2^, 1.5 W/cm^2^, and 2.0 W/cm^2^) for 5 min. (**c**) The photothermal response of IR-CS–PPy NCs (125 μg/mL) aqueous solution exposed to an 808-nm laser source at 2 W/cm^2^ for 300 s and then the laser was shut off for about 900 s. (**d**) Temperature profiles of IR-CS–PPy NCs (125 μg/mL) aqueous solution for five on/off cycles. (**e**) The corresponding NIR thermographic images of the well containing IR-CS–PPy NCs (125 μg/mL) during 300 s of laser irradiation (2 W/cm^2^).

After 5 min of the NIR irradiation (2.0 W/cm^2^), the temperature increased to 28.7 °C for the control group without any IR-CS–PPy NCs materials, whereas the temperature reached 62.6 °C for the IR-CS–PPy NCs (100 µL) inoculated solution for at the highest concentration of 125 µg/mL (Fig. 4a). As shown in Fig. 4b, the temperature of IR-CS–PPy NCs solution (125 μg/mL) increased to 25.7, 35.2, 51.5, and 62.6 °C when the laser power densities were applied at 0.5, 1.0, 1.5, and 2.0 W/cm^2^, respectively. The graph in Fig. 4c and thermal images in Fig. 4e demonstrate the gradual increase of the temperature during the turn-on cycle, whereas a steady decrease after the laser was turned off. The highest possible temperature (62.6 °C) above hyperthermia indicates the promising potential for killing tumor cells during in vivo cancer therapy.

The laser on–off cycle was repeated 5 times to determine the photostability of IR-CS–PPy NCs. As illustrated in Fig. 4d, there is no significant difference in the thermal profile among five cycles of the IR-CS–PPy NCs heating and cooling period. The photothermal conversion efficiency (η) of PTT nanomaterials is also an essential characteristic that has to be verified. Based on the in vitro photothermal data, the linear fitting of the cooling period was performed to define $\tau_{s}$ with a value of 156.48 s (Fig. S9). From the equations in Supporting Information, the photothermal conversion efficiency (η) was calculated as 20.29%, similar to that of conventional photothermal agents such as gold nanorods (~ 21%) and higher than that of Cu_2−x_S nanocrystals (~ 16.3%). The in vitro photothermal results suggest that IR-CS–PPy NCs have consistent photothermal stability and a decent photothermal conversion ability.

### In vitro cell cytotoxicity assay and photothermal therapy

**Figure 5:**
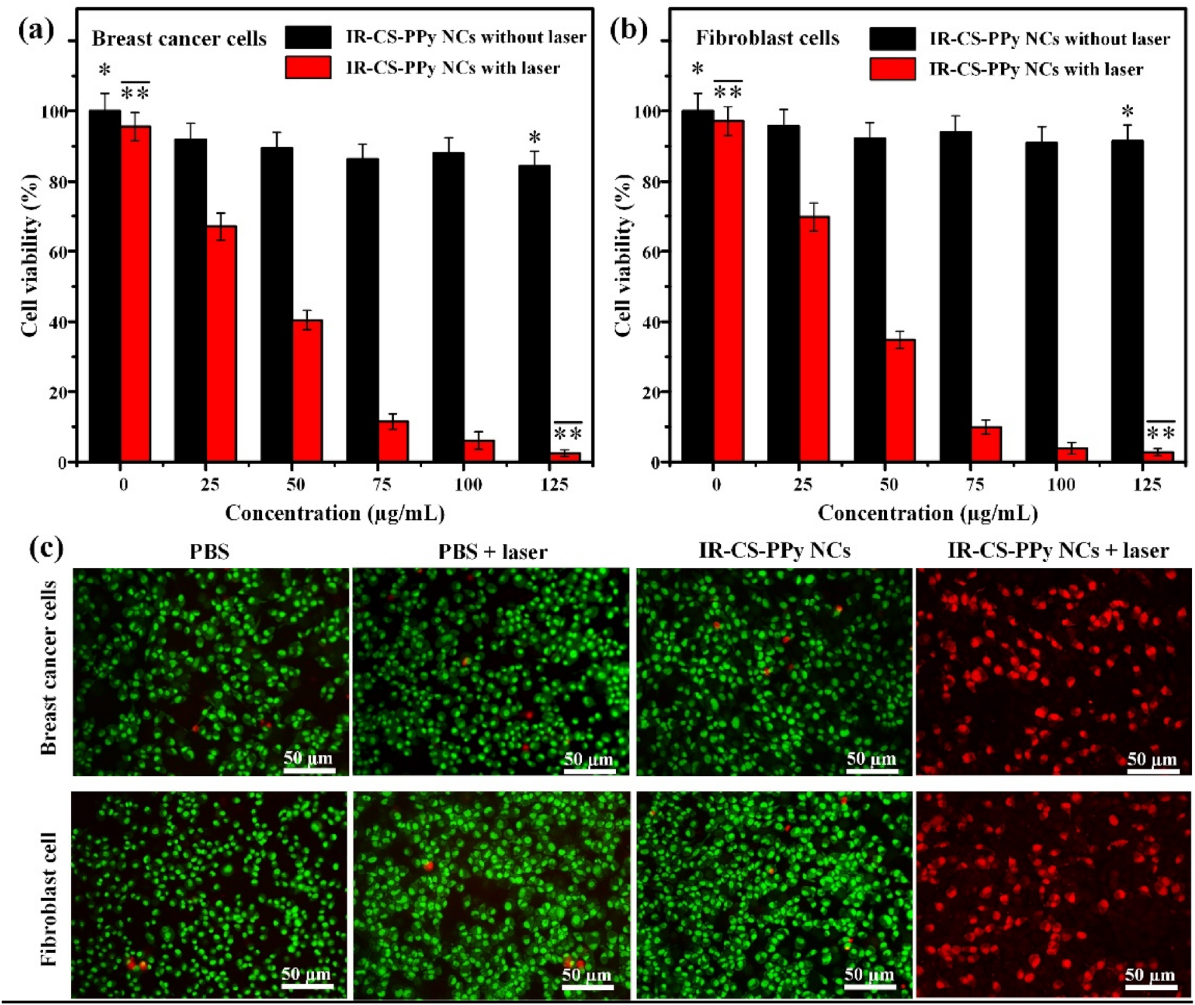
The cell viability of (**a**) MDA-MB-231 breast cancer and (**b**) L929 human normal fibroblast cells treated with IR-CS–PPy NCs with and without NIR laser (2 W/cm^2^, 5 min). (**b**) The cell viability of L929 cells treated with IR-CS–PPy NCs with and without NIR laser (2 W/cm^2^, 5 min). (**c**) AO/PI staining of MDA-MB-231 cells and L929 cells treated with PBS, PBS + laser (2 W/cm^2^, 5 min), 125 µg/mL IR-CS–PPy NCs, and 125 µg/mL IR-CS–PPy NCs + laser (2 W/cm^2^, 5 min). Data are shown as the mean ± standard deviation (n = 3). (*Significant *p* < 0.05).

The biocompatibility of nanomaterials is an essential aspect regarding biomedical applications. The standard MTT assay was used to inspect the cytotoxicity of IR-CS–PPy NCs against MDA-MB-231 breast cancer and L929 human normal fibroblasts cell lines. After 24 h of incubation, there is a slight reduction in the number of living cells following the increase of the IR-CS–PPy NCs dosage (Fig. 5a, b). The cell viability results indicate the good biocompatibility of IR-CS–PPy NCs because no substantial cytotoxicity was detected even at the highest concentration of 125 µg/mL.

The photothermal effect of IR-CS–PPy NCs was inspected based on the viability of MDA-MB-231 breast cancer and L929 human normal fibroblasts cells during the in vitro PTT treatment. The cells were incubated with different concentrations of IR-CS–PPy NCs (0, 25, 50, 75, 100, and 125 μg/mL) and then exposed to 808 nm NIR (2 W/cm^2^) for 5 min. According to the viability studies, IR-CS–PPy NCs coupled with NIR laser could effectively destroy cells in a concentration-dependent manner (Fig. 4b). After being checked using the MTT assay, the photothermal efficiency of IR-CS–PPy NCs was further examined by the fluorescence method. The four groups of MDA-MB-231 and L929 human normal fibroblasts cells were formed based on corresponding treatments: group I (PBS); group II (PBS + NIR laser); group III (CS–PPy NCs, 125 μg/mL); group IV (IR-CS–PPy NCs, 125 μg/mL + NIR laser). Fluorescence dye AO and PI were co-stained with treated cells in every group. AO enters live/ dead cells and emits green fluorescence, whereas, PI only penetrates dead cells and generates red fluorescence. As illustrated in the merged fluorescence images, a few red-fluorescence signals were detected in groups I, II, and III, suggesting the minority of deceased cells. Meanwhile, a significant number of cells in group IV fluoresced red for both types of cell lines, suggesting that most of the cells were ablated (Fig. 5c). The in vitro thermal results conclude that the IR-CS–PPy NCs with the supportive NIR laser could productively enhance the ablation of breast cancer cells. There are no fluorescence hindrances or interference observed for conjugated IR783 dye due to its different excitation and emission wavelength.

### Fluorescence imaging and performance test

Different companies around the world support several commercial fluorescence imaging systems for biomedical applications. The FOBI of Neoscience was used by Chuang Gao et.al. to deal with indocyanine green fluorescence dye^50^. Lokesh Basavarajappa et. al. handled the Pearl Triology from LI-COR Biosciences to detect IR-780 dye^51^. IVIS Spectrum from PerkinElmer is the most popular fluorescence imaging system to monitor the whole mice body during in vivo fluorescence study^52^. The typical fluorescence imaging instruments discussed were used and confirmed by numerous researchers worldwide. But those highly expensive systems are not easily available to most researchers in this field. The price range of these instruments varies from 18,000$ to 550.000$ and more depending upon functions and applications. The goal of our study is to fabricate a fluorescence system at an affordable price without compromising imaging quality. The lateral resolution of the proposed fluorescence imaging system was measured using a USAF resolution test chart (R3DL3P, Thorlabs, Newton, NJ, USA) (Fig. S1a, S1b). From the equations in Supporting Information, the lateral resolution was calculated as 140.29 μm following the previously reported method^53^. The different concentrations of IR-CS–PPy NCs (0, 25, 50, 75, 100, 125 μg/mL) were used to calculate the signal-to-noise ratio (SNR) of the fluorescence system. The SNR got the peak of 40.29 at the highest concentration (125 μg/mL). The higher concentration of IR-CS–PPy NCs (25, 50, 75, 100 μg/mL) enhanced the SNR of the fluorescence system at 5.74, 12.6, 21.91, 36.61, respectively (Fig. S1c).

**Figure 6:**
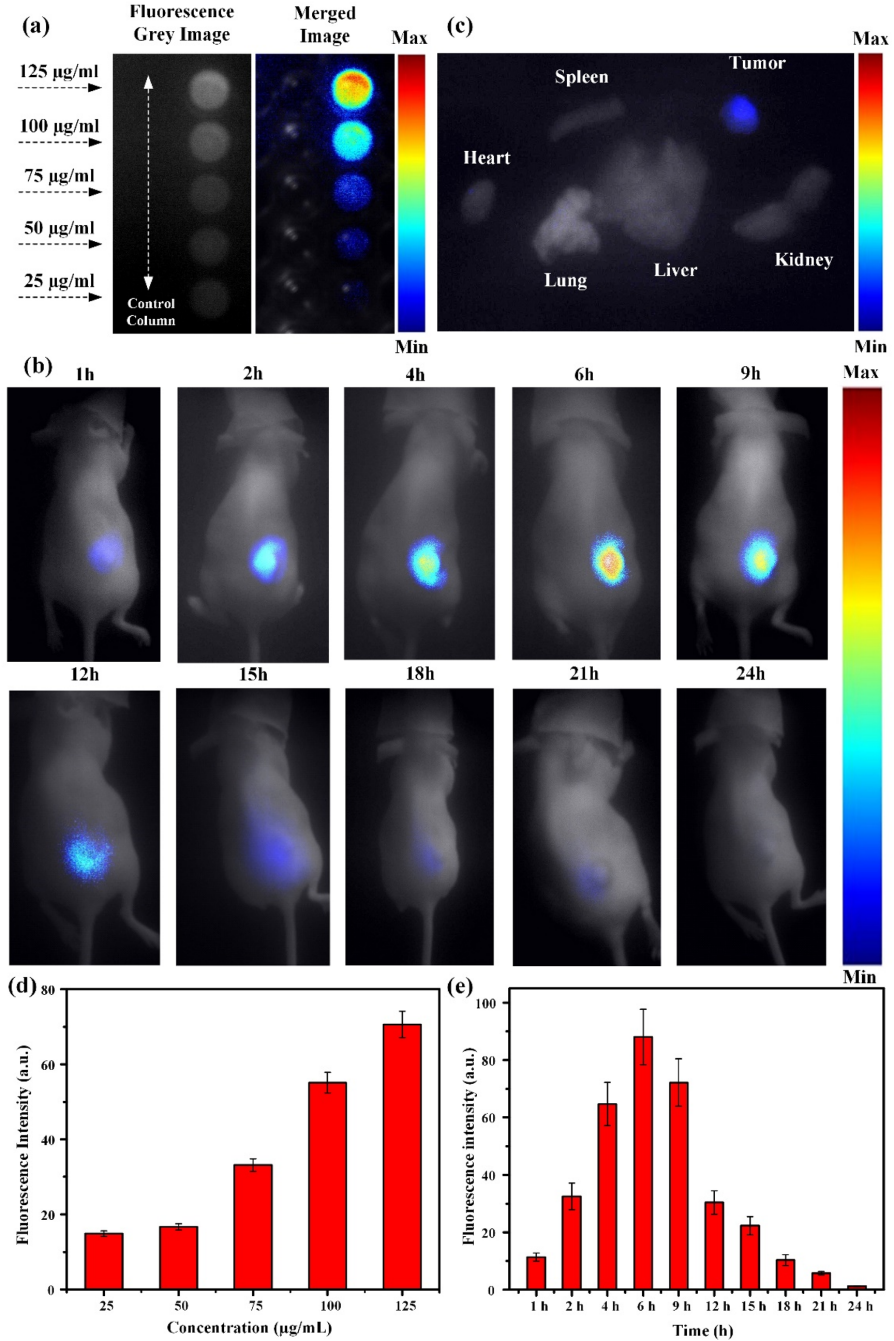
(**a**) In vitro fluorescence imaging of control cells and MDA-MB-231 cell treated with different concentrations of IR-CS–PPy NCs (25, 50, 75, 100, and 125 μg/mL) (**b**) In vivo fluorescence imaging of MDA-MB-231 tumor-bearing nude mice at different time points after local injection of IR-CS–PPy NCs. (**c**) Ex vivo fluorescence imaging of the organs including, the heart, liver, lung, kidney, and spleen, and tumors excised from the mice treated with IR-CS–PPy NCs at 24 h post local injection. (**d**) The mean fluorescence intensities of different concentrations of IR-CS–PPy NCs were measured, displaying the highest fluorescence intensity at 125 μg/mL. (**e**) The mean fluorescence intensities of tumors were quantified at different time points, showing a peak value after 6 h injection of IR-CS–PPy NCs. Data are shown as the mean ± standard deviation (n = 3). Min = 0, and Max = 100.

For in vitro fluorescence study, two columns of dual control/fluorescent dye groups were prepared after several steps. In Fig. 6a, no fluorescence signal was detected in five control wells while the strong fluorescence intensity at different concentrations of IR-CS–PPy NCs was identified. Among them, the strongest one was observed at the 125 μg/mL concentration of IR-CS–PPy NCs (Fig. 6d). After verifying in vitro, the highest concentration (125 μg/mL) of IR-CS–PPy NCs was locally injected into the mice tumor for checking the in vivo distribution using the NIR fluorescence imaging system. The fluorescence images of tumor-bearing mice were captured at different time points (1, 2, 4, 6, 9, 12, 15, 18, 21, and 24 h) until no evidence of IR-CS–PPy NCs was observed (Fig. 6b). Right after intratumoral injection, few fluorescence signals were detected due to the attenuation of IR783 in water^54^. Next, the IR-CS–PPy NCs were slowly accumulated by the tumor cells while the injected water solution dissipated from the treated area. Therefore, the fluorescence intensity gradually increases and reached a peak after 6 h. After that, it slowly decreases until 24 h due to the metabolic activity of the cellular system (Fig. 6e). The intensity peak indicated the moment of strongest activity of IR-CS–PPy NCs inside the tumor area and proved that 6 h after injection is the ideal time for the in vivo photothermal therapy. Furthermore, the distribution of IR-CS–PPy NCs was investigated through ex vivo fluorescence imaging. After 24 h of injection, the mice used for study in vivo were euthanized to collect and analyze organs and tumors. As observed in the customized NIR fluorescence system, the fluorescence signal was only observed in tumor, whereas no significant signal was found in other organs (Fig. 6c).

### Photoacoustic imaging and performance test

The PAI system was widely used to spot the distribution of nanoparticles inside the mice body due to their high-resolution detection characteristics. Several studies were reported showing high-quality images of nanoparticles distribution during in vivo experiment^55,56^. Our PAI system was presented for the sake of collecting the same level of image quality. The characterization of the proposed PAI system was conducted using 6-μm width carbon fiber, black tape and chicken breast tissue. The measured lateral and axial resolutions were 7 μm and 75 μm, respectively (Fig. S2a and S2b). The longest distance from the tissue surface to visible black tape (Fig. S2c) indicating the penetration depth (~ 1.2 mm) of our PAI system. Fig. S2c demonstrates the SNR of the PAI system which increased from 8.33 to 12.5 when the IR-CS–PPy NCs concentration increased from 75 to 125 μg/mL. (See Supplementary Information for more details about resolution, penetration depth, SNR calculation of PAI system).

**Figure 7:**
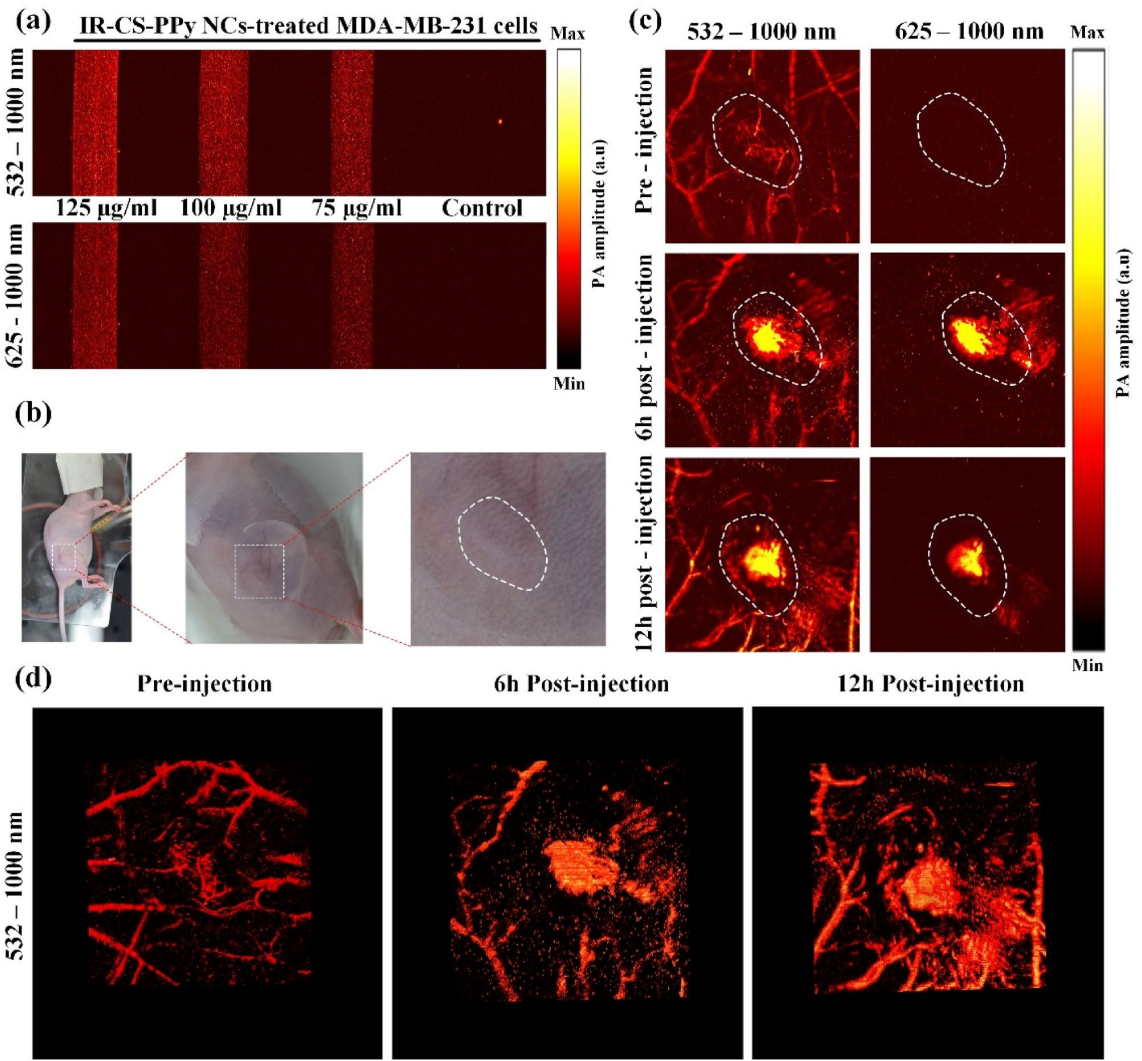
(**a**) In vitro PAI of MDA-MB-231 cells incubated with various concentrations of IR-CS–PPy NCs using PAI system with 532–1000 nm wavelength and 625–1000 nm wavelength. (**b**) Representative digital photographs of MDA-MB-231 tumor-bearing nude mice for in vivo PAI. The white dash lines indicate the tumor area. (**c**) In vivo PAI of tumor tissues in MDA-MB-231 tumor-bearing nude mice at 0, 6, and 12 h after injection of IR-CS–PPy NCs (100 μL) using PAI system with 532–1000 nm wavelength, and 625–1000 nm wavelength. (**d**) Representative 3D PA images of tumor tissues MDA-MB-231 tumor-bearing nude mice at 0, 6, and 12 h after injection of IR-CS–PPy NCs using PAI system with 532–1000 nm wavelength.

The performance of the fabricated PAI system was further tested with in vitro and in vivo experimental studies. After 6 h of incubation with different concentrations of IR-CS–PPy NCs (75, 100, and 125 μg/mL), the MDA-MB-231 cells were harvested. Thus, after trypsin digestion, the cells were pumped into PTFE tubes and used for in vitro PAI study (Fig. S3). The three tubes containing IR-CS–PPy NCs-treated cells emitted signals; meanwhile, no PA signal was produced from the control tube (Fig. 7a). The IR-CS–PPy NCs (highest concentration at 125 μg/mL) produced a higher PA signal at 532–1000 nm and 625–1000 nm compared to the lower ones (Fig. S4). The in vitro results indicate the IR-CS–PPy NCs’ ability as a PAI-guided nanomaterial.

For the very first of in vivo PAI, the tumor area of MDA-MB-231-bearing mice was scanned using the PAI system with laser (wavelength: 532–1000 nm) to visualize the local microvascular system. Followed by a long-pass filter was used to block the wavelength under 625 nm for the sake of removing the PA image of blood vessels. As illustrated in Fig. 7c, in the pre-injection part, the blood vessels gather at a higher density at the tumor area in the 532–1000 nm wavelength, whereas all blood vessel images were blocked by the filter in the 625–1000 mm wavelength. Next, 0.1 mL IR-CS–PPy NCs (125 μg/mL) was prepared and locally injected into the tumor area of MDA-MB-231-bearing mice (Fig. 7b). After 6 h and 12 h of injection, the same process as pre-injection was repeated. The tumor area of injected mice generated a higher PA signal due to the appearance of IR-CS–PPy NCs in the 532–1000 nm wavelength at 6 h. The presence and extension of IR-CS–PPy NCs were clearly revealed with support from the filter in the 625–1000 nm image. Because of the circulation of blood during mice’s activities, the PA intensity of the IR-CS–PPy NCs was fairly reduced after 12 h of injection (Fig. S5). The in vivo PAI results prove the optimal time for the photothermal therapy was 6 h post-injection of IR-CS–PPy NCs. The reconstructed 3D images in Fig. 7d and Video S1, S2, and S3 clarify the distribution of injecting nanomaterials inside the mice samples.

The proposed PAI system constructed a high-contrast tumor structure and nanomaterial distribution with a high spatial resolution. The additional IR-CS–PPy NCs enhanced the PA intensity and served as an effective PA contrast agent for following photothermal therapy. Similar results have been presented by Manivasagan et al.^15^ Thi Tuong Vy, Phan et al.^27^.

### In vivo photothermal therapy

**Figure 8:**
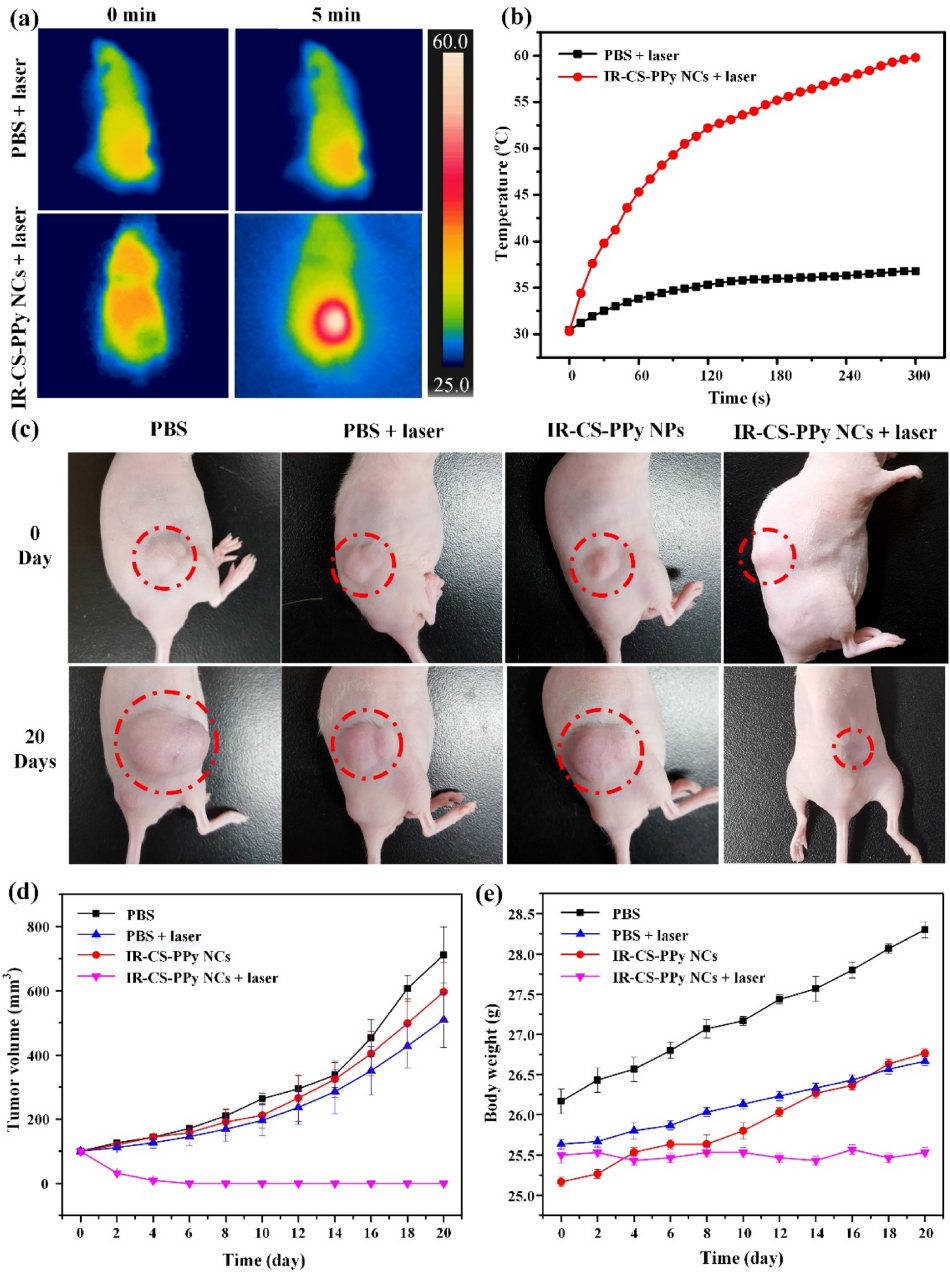
(**a**) NIR thermographic images of tumor-bearing mice with intratumoral injection of PBS and IR-CS–PPy NCs with 808-nm NIR laser irradiation at 2.0 W/cm^2^ for 5 min. (**b**) Temperature change of tumor-bearing mice after intratumoral injection of with IR-CS–PPy NCs with 808-nm NIR laser irradiation at 2.0 W/cm^2^ for 5 min. (**c**) The digital photographs of tumor-bearing mice taken at day 0 before treatment and 20 days after treatment. (**d**) Tumor volume growth curves of different groups of mice after different treatments. Data presented as mean ± standard deviation. (n = 3). (**e**) The body weight after different treatments indicated in 20 days. Data are shown as the mean ± standard deviation (n = 3).

To further investigate the thermal efficiency of IR-CS–PPy NCs, in vivo PTT study was performed on tumor-bearing mice. The thermal image of treated mice was captured using the i5 IR thermal camera to monitor the temperature variation in tumor areas. As illustrated in Fig. 8a and b, the tumor temperature of group IV under the effect of the IR-CS–PPy NCs and NIR laser, reached 59.8 °C within 300 s, whereas the only PBS without any nanoparticle raised up to 36.8 °C temperature when irradiated with 808-nm laser for 300 s. The temperature peak resulted in by IR-CS–PPy NCs-assisted laser was able to induce the tumor ablation in vivo. No sudden death during laser treatment was witnessed, and no considerable weight change was recorded on daily observation basis (Fig. 8e). The overall experimental study proves that IR-CS–PPy NCs are highly biocompatible and did not produce any considerable toxicity in vivo due to their photothermal impact. As illustrated in Fig. 8d, there were negligible differences in tumor volumes and tumor growth rates in groups I, II, and III, suggesting that NIR laser irradiation (808 nm) and IR-CS–PPy NCs individually did not significantly affect tumor growth. Meanwhile, the IR-CS–PPy NCs-mediated PTT could effectively retard tumor growth and completely destroy tumors after 20 days (Fig. 8c). After successful treatment, only scars were found at the tumor site; thus, the treated mice in all groups were euthanized by cervical dislocation to collect all organs and tumors (Fig. S6). As illustrated in Fig. S7, the tumors for all groups were collected, weighed, and compared. There was no tumor found for group IV mice; which indicates the efficiency of IR-CS–PPy NCs that generates sufficient heat to ablate the tumor without regrowth. Histological analysis (Fig. S8) also revealed no noticeable toxic effect of IR-CS–PPy NCs on the major organs of mice after 20 days of photothermal treatment. Comparing with the control group, no significant tissue morphological differences were observed for IR-CS–PPy NCs treated group of animals. In case of the control group, the histological analysis for tumor sample, densely packed tumor cells were identified with deep staining. Whereas, IR-CS–PPy NCs treated group of mice was successfully cured and no tumor was observed.

## Conclusion

In this present study, the improved IR-CS–PPy NCs were synthesized as multimodal imaging-guided PTT contrast agents. Because of the great NIR absorption, the proposed IR-CS–PPy NCs exhibited high-efficiency therapeutic capacity and thermal stability, which were demonstrated during in vitro thermal therapy. The synthesized NPs were also highly biocompatible, as there was no significant decrease in the number of live cells when treated with different concentrations. After injecting the nanocomposites at the tumor areas, the targeted regions were diagnosed very accurately by the FL/PA imaging systems. The fluorescence and photoacoustic modules were proposed and fabricated to acquire the experimental images during the imaging period of in vitro, in vivo*,* and ex vivo studies. After 20 days of the treatment with IR-CS–PPy NCs with NIR laser irradiation, the treated tumors disappeared and the MDA-MB-231 tumor-bearing mice fully recovered without noticeable side effects. Generally, our findings demonstrate that the multimodal imaging system based on the proposed nanomaterials is capable of effectively treating the tumors in vivo. The non-toxic and stable characteristics along with thermal and dual-modal imaging properties of the synthesized IR-CS–PPy NCs could be a promising theragnostic agent for biomedical applications.

## Materials and methods

### Synthesis of CS–PPy NCs and IR-CS–PPy NCs

Based on the earlier reported protocol, we developed the chitosan–polypyrrole nanocomposites (CS–PPy NCs)^57^. In brief, at room temperature, 0.15 g CS was added and stirred in 30 mL of 0.25% acetic acid solution until it had completely dissolved. Next, 0.90 g FeCl_3_·6H_2_O was dissolved in the CS aqueous solution. After that, the above solution was slowly added with 100 μL of pyrrole in a 0 °C–5 °C chilled water bath. The solution rapidly turned black, indicating the CS–PPy NCs formation, and was stirred for another hour to be a uniform solution. The unexpected iron ions were carefully removed by using a dialysis tube (2000 MWCO, Sigma–Aldrich Co., St. Louis, MO, USA). The purified CS–PPy NCs were able to be used for further characterization. Finally, the IR783—fluorescence dye—was conjugated with synthesized CS–PPy NCs as followed by the previously reported method by Qianhue Feng et al.^58^.

### Characterization of synthesized CS–PPy NCs and IR-CS–PPy NCs

The absorption spectrum of CS–PPy NCs and IR-CS–PPy NCs was discovered using UV–Vis spectroscopy (Thermo Biomate 5 Spectrophotometer). The structure of the synthesized CS–PPy NCs was studied through X-ray diffraction (XRD) patterns in the step-scan mode using a Philips X'Pert-MPD PW 3050 diffractometer with Cu K_α_ (40 kV, 30 mA). Fourier transform infrared spectroscopy (FTIR, Perkin Elmer Inc., USA) and Raman spectroscopy (Horiba Jobin Yvon RAM HR800) with frequencies ranging from 4000 to 400 cm^−1^, were used to investigate the functional and structural groups. The CS–PPy NCs and IR-CS–PPy NCs' size and composition were characterized using field emission transmission electron microscopy (FE-TEM; JEM-2100F, JEOL, Japan). The working condition for FE-TEM was maintained as follows: TEM lattice resolution at 200 kV was 0.1 nm, with probe current: 2.5 nA at 0.7 nm of probe diameter (pressure 1 × 10^−8^ Pa).

### In vitro cell culture study

In this study, the breast cancer cell line (MDA-MB-231) and human normal fibroblasts (L929) cell line were used to assess the ability for biomedical application of the proposed IR-CS–PPy NCs. The standard DMEM media used to culture the cells procured from the Korean Cell Line Bank (KCLB, Seoul, Republic of Korea). In a humidified 5% CO_2_ incubator, the cells were grown at 37 °C for the entire experimental study.

### The animal model

All the animal experiments were performed in accordance with the animal ethical committee guidelines and regulations approved by Pukyong National University, Busan, Republic of Korea All the animal experimental studies were approved by ethics committee of Pukyong National University, Busan, Republic of Korea. The protocol PKNU IACUC-2019-09 for the animal experiment study was strictly followed and performed following ARRIVE guidelines and regulations for the care and use of laboratory animals. Six-week-old BALB/c female nude mice (~ 20 g) purchased from Orient Bio Inc. (Seongnam, Republic of Korea) were used for in vivo experiments regarding the institutional guidelines. For the animal cancer model, 100 μL phosphate-buffered saline (PBS) suspension of 1 × 10^6^ MDA-MB-231 cells was subcutaneously injected into the right flank of all the mice. The dimensions of tumors were measured every day using a digital caliper until their volume reached approximately 100 mm^3^.

### Fluorescence imaging system design (LUX 3.0)

All of the fluorescence images in this study were obtained from the indigenously developed NIR fluorescence imaging system (LUX 3.0). A self-made controller printed circuit board (PCB) was designed to manipulate the whole system. There are several blocks in this PCB with the 8-bit microcontroller (PIC16F1713-I/SS, Microchip Technology Inc., Austin, TX, USA) being used for the primary processor cores. The microcontroller set the output current of the light-emitting diode (LED) driver (RCD-24-1.2, RECOM Power, Dietzenbach, Germany) through the digital-analog converter mode. Every LED whose working current is smaller than 1.2 amp can be connected with this driver. An indigenous motorized filter wheel was fabricated consisting of a step motor (42HS4013A4G18, NEMA, Arlington, VA, USA), a motor driver (DRV8825, Texas Instruments, Dallas, TX, USA), and a proximity sensor (LJ8A3-2-Z/BX-5V, Suzhou Leoho Electronics, Suzhou, China). The chamber was lightened up by the white LED module (LMMW1, OMC Ltd., Redruth, UK) to obtain the background image. The user interface was built based on a 5-inch liquid crystal display touchscreen (NX8048T050, Nextion, Shenzhen, GD, China) with the supportive Nextion Editor programming software.

**Figure 9:**
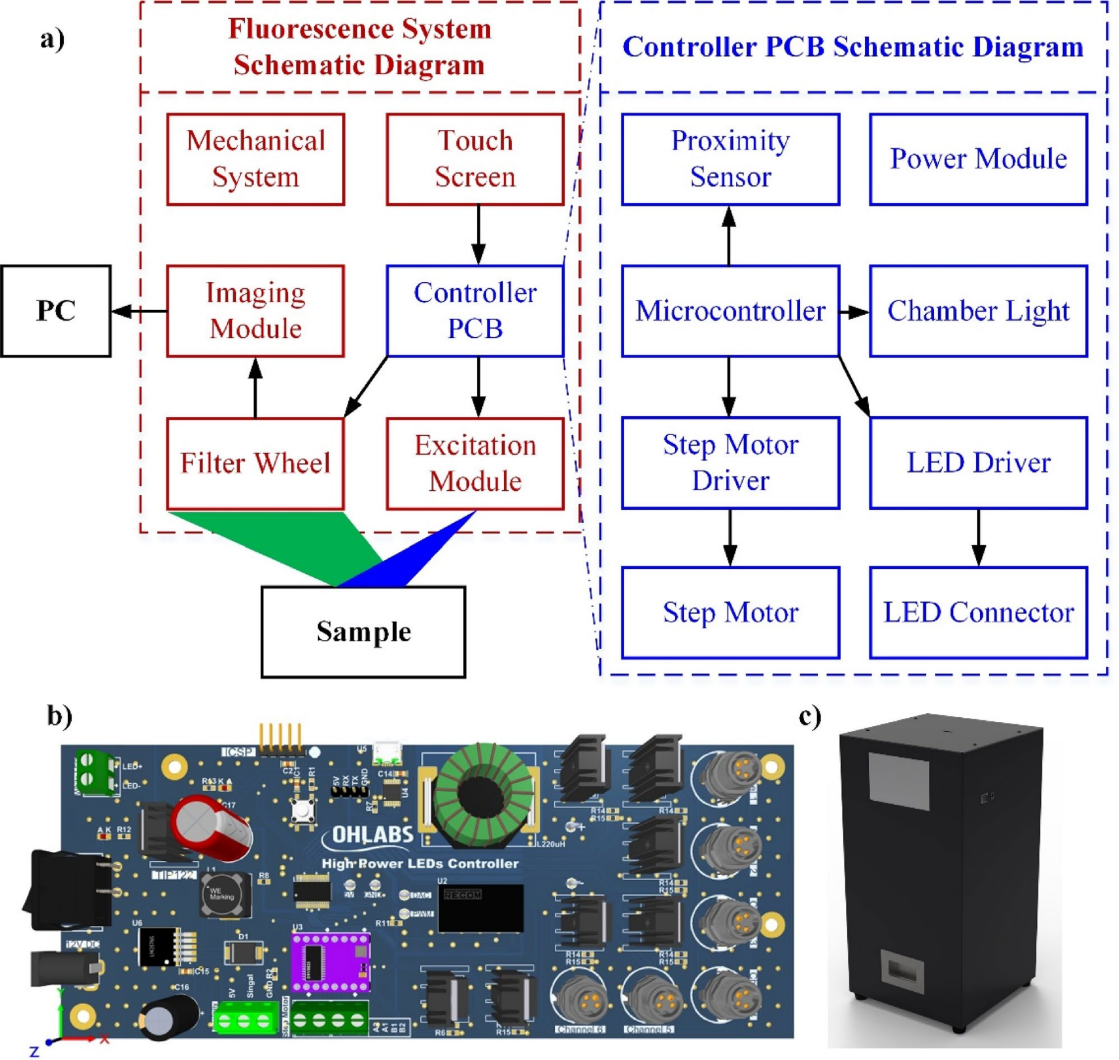
The proposed fluorescence imaging system (**a**) Schematic diagram of fluorescence system and the controller printed circuit board (PCB) (**b**) 3D design of controller PCB (**c**) 3D design of fluorescence system.

For dealing with the IR783 fluorescence dye, we used a compact excitation module including a 780-nm NIR LED (M780L3; Thorlabs, Newton, NJ, USA) and a required excitation filter (FF01-769/41-25; Semrock, Rochester, NY, USA). The output light from the LED was justified by a special coating collimated lens (ACL2520B; Thorlabs, Newton, NJ, USA) to effectively irradiate the sample. Another corresponding filter (FF01-832/37-25; Semrock, Rochester, NY, USA) was used to minimize the background noise of emission signals generated from the fluorescence contrast agent. To acquire all filtered fluorescent images, an imaging module was built including a USB 3.0 NIR camera (GS3-U3-41C6NIR-C; FLIR Systems, Wilsonville, OR, USA) integrated with a machine vision lens (V1628-MPY 1.1 f/2.8; CBC Group, Phoenix, AZ, USA). The image acquisition was performed using the FlyCapture (FlyCapture Software Development Kit (SDK) 2.0; FLIR Systems, Wilsonville, OR, USA) and the acquired images were processed by ImageJ (ImageJ 1.53e; National Institute of the Health, MD, USA). Figure 9a is a schematic diagram of the fluorescent system. The 3D model of the controller PCB and the fluorescence system are presented in Fig. 9b and c, respectively.

### Photoacoustic imaging system design

**Figure 10:**
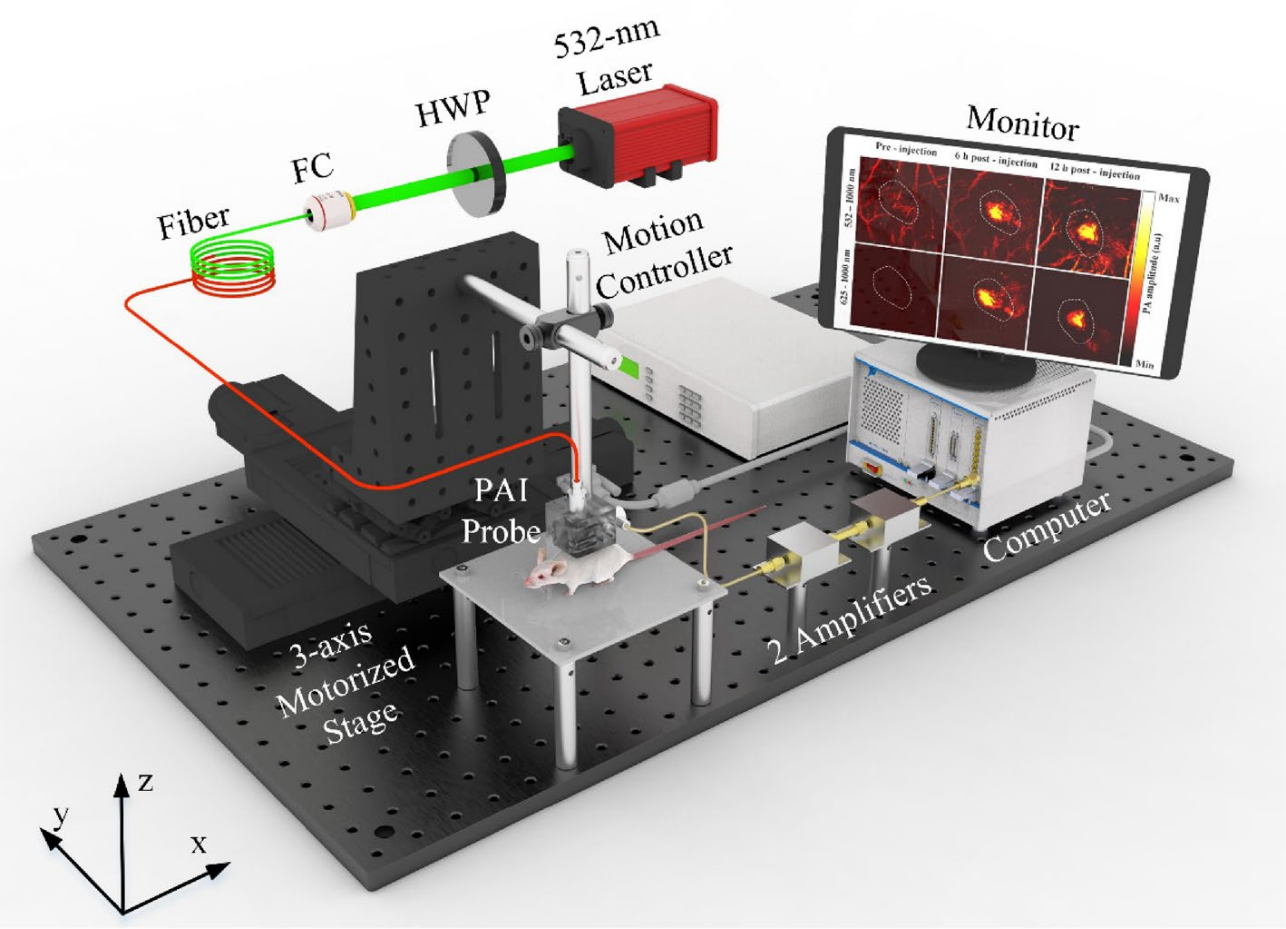
Schematic of the proposed photoacoustic imaging system. *HWP* half-wave plate, *FC* fiber coupler.

In the current study, we designed and developed a customized high-resolution PAI system whose schematic diagram is represented in Fig. 10. A high-efficiency diode-pumped Q-switched 532-nm laser (SPOT-10-100-532; Elforlight, Daventry, UK) was used at a 5 kHz frequency to excite the system. The generated light was coupled into a 2 m single-mode patch cable (P3-460B-FC-2; Thorlabs, Newton, NJ, USA) by a half-wave plane and a fiber coupler. The coupled laser output was filtered and collimated by several objective lenses and a band-pass filter to achieve near-diffraction-limited laser focus. The laser energy density adjusted to be under 13.3 mJ/cm^2^ was utilized to irradiate the sample. A tailored-made PAI probe was fabricated to guide the laser light and maximized the generated ultrasound waves in the reflecting mode as followed from a previous study^59^. The acoustic signals were received by an attached 25 MHz transducer with a 0.5-inch focal length (V324-SM; Olympus, Norfolk, VA, USA). A z-axis stage was used to synchronize the laser spot and transducer focal point to gain the optimum ultrasound signals, and a 2D motorized stage (x- and y-axis) was used to scan the sample. The focused transducer was then connected with two serial preamplifiers (ZFL-500LN; Mini-Circuits, Brooklyn, NY, USA) to boost the received signal before transmitting it to the acquisition system (NI PXI-5124; National Instruments, Austin, TX, USA). The electrical trigger created at the 10 µm spatial step of the linear actuator was synchronized with the laser to construct the 2D PA image. The system had to operate for approximately 10 min to scan an 8 × 8 mm field-of-view image. Finally, an open-source software platform (3D Slicer version 4.10.2; slicer.org) was used to build the 3D model.

### Fluorescence imaging

The MDA-MB-231 cells were incubated with IR-CS–PPy NCs for different concentrations (5, 25, 50, 100, and 125 μg/mL) and were ready for the in vitro study. The fluorescence intensities of IR-CS–PPy NCs were observed within 6 h. For the in vivo fluorescence, the IR-CS–PPy NCs (125 μg/mL) was locally injected into the MDA-MB-231 tumor-bearing mice. The in vivo fluorescence distribution at different time intervals (1, 2, 4, 6, 9, 12, 15, 18, 21 and 24 h) was monitored using the customized fluorescence imaging system described earlier. After the imaging period, the mice were euthanized by carbon dioxide inhalation followed by cervical dislocation to collect all organs including the liver, lung, kidney, spleen, heart, and tumors. The ex vivo fluorescence was performed after washing all of the obtained organs with cold PBS.

### Photoacoustic imaging

The previously described PAI system was used to conduct the photoacoustic imaging experiment. Various concentrations of IR-CS–PPy NCs NCs (75, 100, and 125 μg/mL) were used to treat the MDA-MB-231 breast cancer cells for 6 h. The treated cells were centrifuged for 3 min at 950 rpm after trypsin digestion. The polytetrafluoroethylene (PTFE) tubes were prepared to contain the cell pellets as the in vitro PAI sample. The IR-CS–PPy NCs with 125 μg/mL concentration was selected to use for in vivo PAI. The prepared solutions were applied to the right flank of tumor-bearing nude mice through intratumoral injection. The in vivo PA images of IR-CS–PPy NCs distribution were constructed at pre-injection, 6 and 12 h post-injection.

### Measurement of the photothermal performance of IR-CS–PPy NCs

The IR-CS–PPy NCs were dispersed in distilled water at various concentrations (0, 25, 50, 75, 100, and 125 µg/mL), followed by inoculation of 1 mL prepared solution to each 12-well plate for assessing photothermal performance. Each well was directly exposed to 808-nm NIR laser at 2 W/cm^2^ of power density for 5 min. The well with the highest concentration (125 µg/mL) was further irradiated with different power densities (0.5, 1, 1.5, 2 W/cm^2^) for 5 min. The real-time temperature of the exposed area was monitored using an infrared (IR) thermal camera (FLIR i5, FLIR Systems, Wilsonville, OR, USA) and a digital thermometer.

The photothermal stability of IR-CS–PPy NCs was studied using a quartz cuvette. The 1 mL aqueous solution of the IR-CS–PPy NCs (125 μg/mL) was applied to the quartz cuvette irradiated by 808-nm NIR laser for 300 s at a density of 2 W/cm^2^. The temperature fluctuations were measured during five cycles of laser activation accompanied by five cycles of heating, cooling process. According to previously reported studies^60,61^, the photothermal conversion efficiency (η) was obtained (see Supporting Information).

### In vitro cytotoxicity assay and photothermal efficiency

The percentage of viable MDA-MB-231 breast cancer cells and L929 human normal fibroblasts cell lines was calculated to evaluate the cytotoxicity of IR-CS–PPy NCs. The 96-well plates were used for seeding the cells at a concentration of 1 × 10^4^ cells/well. After 24 h of incubation with different concentrations of IR-CS–PPy NCs (0, 25, 50, 75, 100, and 125 µg/mL), 100 µL MTT was poured in each treated well to assess the cell viability. To evaluate the in vitro photothermal efficiency of IR-CS–PPy NCs, another group of IR-CS–PPy NCs-treated cells was irradiated with 2 W/cm^2^ NIR laser for 5 min. Next, the cells were incubated for a further 2 h, and finally, the MTT assay was performed to quantify the cell viability post-NIR laser treatment.

For qualitative photothermal capacity evaluation, 12-well plates were used to incubate MDA-MB-231 and L929 cells (2 × 10^5^ cells per well) for 24 h at 37 °C of temperature. After removing the culture media, we separate the cells into four groups: group I (PBS treatment), group II (PBS plus NIR laser treatment), group III (125 μg/mL IR-CS–PPy NCs treatment), and group IV (125 μg/mL IR-CS–PPy NCs plus NIR laser treatment). After 5 min of laser exposure, the cells in all groups were stained with acridine orange (AO) and propidium iodide (PI). The in vitro photothermal effect was assessed based on fluorescence images of the samples obtained using a Leica DMI300B fluorescence microscope (Leica Microsystems, Wetzlar, Germany).

### In vivo photothermal therapy

The in vivo photothermal therapy efficiency of the synthesized IR-CS–PPy NCs was examined using MDA-MB-231 tumor-bearing mice. The mice tumors were grown until their volume reached around 100 mm^3^. After that, the mice were divided into four groups (n = 3): group 1 treated with only PBS, group 2 treated with PBS + NIR laser, group 3 treated with only IR-CS–PPy NCs, and group 4 treated with IR-CS–PPy NCs + NIR. The mice were given 100 µL of PBS or IR-CS–PPy NCs with a concentration of 125 g/mL through intratumoral injection. After 6 h of injection, groups II and IV received thermal therapy through the exposure of the tumor areas to the NIR laser at a density of 2.0 W/cm^2^ for 300 s. The thermal camera was used to continuously monitor the tumor temperature change during treatments. Until 20 days after injection, all mice' weight was checked using lab weighing scales, and tumor volume was measured after every two days interval using a digital caliper. The tumor volume (mm^3^) was calculated using the equation V = (1/2) × (L × W^2^), where, L is the tumor's length, and W is its width.

### Histological analysis

After 20 days of experimental study, five major organs: liver, spleen, kidney, lung, heart and tumor were harvested from the control and treated group of mice. Each organ was dehydrated by neutral buffered formalin and fixed with a series of chemical immersion for histological analysis. The chemically fixed samples were microtomed into 4 μm thick sections for further staining with hematoxylin and eosin (H&E) and observed under the optical microscope.

### Statistical analysis

All the data were expressed as the mean ± standard deviation. Statistical analyses were carried out using one-way analysis of variance. OriginPro 8.0 from OriginLab corp. (Northampton, MA, USA) was used to analyze the data.

## Supplementary Information

Supplementary Information 1.

Supplementary Video S1.

Supplementary Video S2.

Supplementary Video S3.
