# Author Correction: Fluorescence/photoacoustic imaging-guided nanomaterials for highly efficient cancer theragnostic agent

**DOI:** 10.1038/s41598-021-98159-6

**Published:** 2021-09-08

**Authors:** Vu Hoang Minh Doan, Van Tu Nguyen, Sudip Mondal, Thi Mai Thien Vo, Cao Duong Ly, Dinh Dat Vu, Gebremedhin Yonatan Ataklti, Sumin Park, Jaeyeop Choi, Junghwan Oh

**Affiliations:** 1 Industry 4.0 Convergence Bionics Engineering, Department of Biomedical Engineering, Pukyong National University, Busan, 48513 Republic of Korea; 2 New-Senior Healthcare Innovation Center (BK21 Plus), Pukyong National University, Busan, 48513 Republic of Korea; 3 Ohlabs Corp., Busan, 48513 Republic of Korea

Correction to: *Scientific Reports* https://doi.org/10.1038/s41598-021-95660-w, published online 05 August 2021

The original version of this Article contained an error, where the model number “LUX 3.0” for the fluorescence imaging system was omitted.

As a result, in the Materials and Methods section, subsection name:

“Fluorescence imaging system design”,

now reads:

“Fluorescence imaging system design (LUX 3.0)”.

“All of the fluorescence images in this study were obtained from the indigenously developed NIR fluorescence imaging system”.

now reads:

“All of the fluorescence images in this study were obtained from the indigenously developed NIR fluorescence imaging system (LUX 3.0)”.

The original Article has been corrected.

