# Supplementary material for: Fluorescence/photoacoustic imaging-guided nanomaterials for highly efficient cancer theragnostic agent
Source: Sci Rep. 2021 Aug 5;11:15943. doi: 10.1038/s41598-021-95660-w (PMC8342712; doi:10.1038/s41598-021-95660-w)
Supplement: Supplementary file 1 — Supplementary Information 1. [file 41598_2021_95660_MOESM1_ESM.docx]

Fluorescence/photoacoustic imaging-guided nanomaterials for highly efficient cancer theranostics agent

Vu Hoang Minh Doan^1,2,†^, Van Tu Nguyen^1,2,†^, Sudip Mondal^2^, Thi Mai Thien Vo^1,2^, Cao Duong Ly^1^, Dinh Dat Vu^1^, Gebremedhin Yonatan Ataklti^1^, Sumin Park^1,2^, Jaeyeop Choi^1^ and Junghwan Oh^1,2,3,*^

^1^ Industry 4.0 Convergence Bionics Engineering, Department of Biomedical Engineering, Pukyong National University, Busan 48513, Republic of Korea

^2^ New-senior Healthcare Innovation Center (BK21 Plus), Pukyong National University, Busan
48513, Republic of Korea

^3^ Ohlabs Corp., Busan 48513, Republic of Korea

† V.H.M.D. and V.T.N contributed equally.

* Corresponding author:

Prof. Junghwan Oh^1,2,3*^

Email: [jungoh@pknu.ac.kr](mailto:jungoh@pknu.ac.kr) (J. Oh). Tel: +82-51-629-5771; Fax: +82-51-629-5779.

Supplementary experimental section


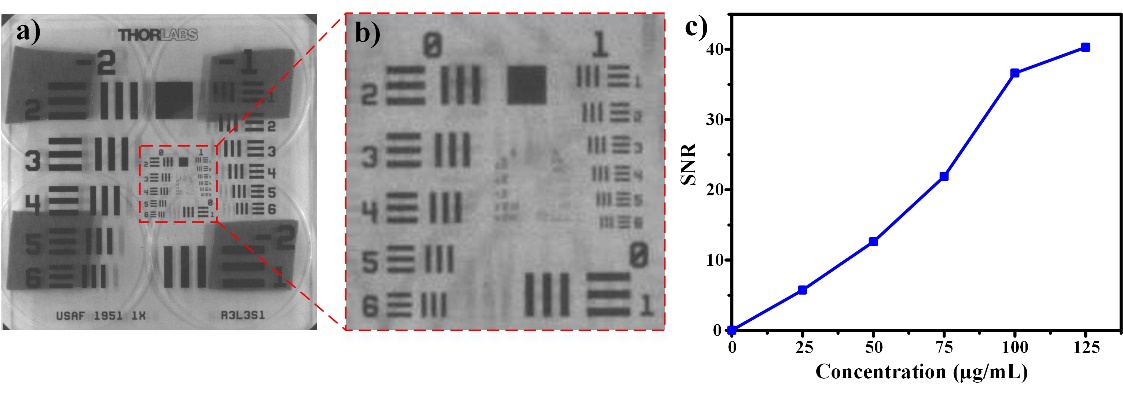


Figure S1. The spatial resolution and SNR of proposed fluorescence system. (a) The image of USAF 1951 resolution test target taken by fluorescence system. (b) Close-up of the red-dash region. (c) Fluorescence signal-to-noise as a function of IR-CS-PPy NCs concentration.


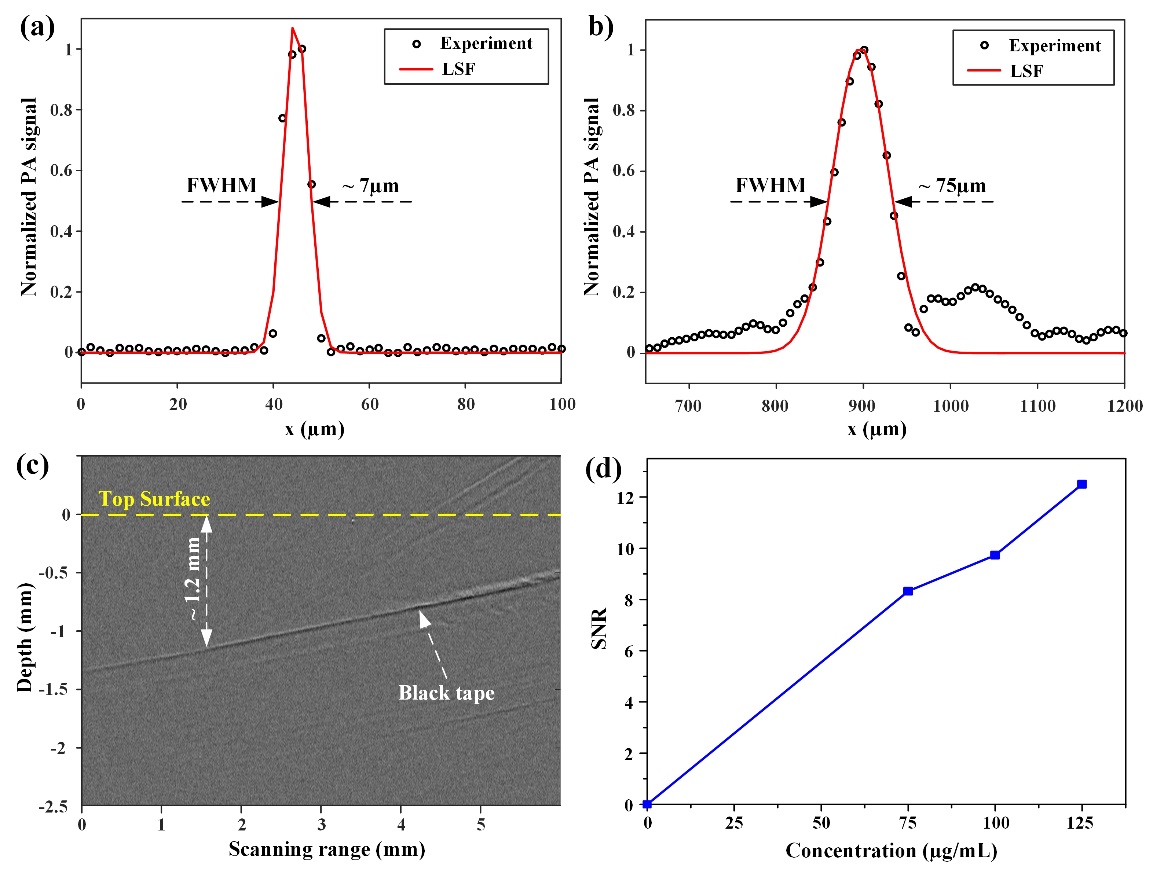


Figure S2. The spatial resolution, penetration depth and SNR of proposed PAI system. (a) The lateral resolution of proposed PAI system. (b) The axial resolution of proposed PAI system. (c) Cross-sectional PA B-scan image of black tape in chicken tissue. (d) PA signal-to-noise as a function of IR-CS-PPy NCs concentration. LSF: line spread function, FWHM: full width at half maximum.


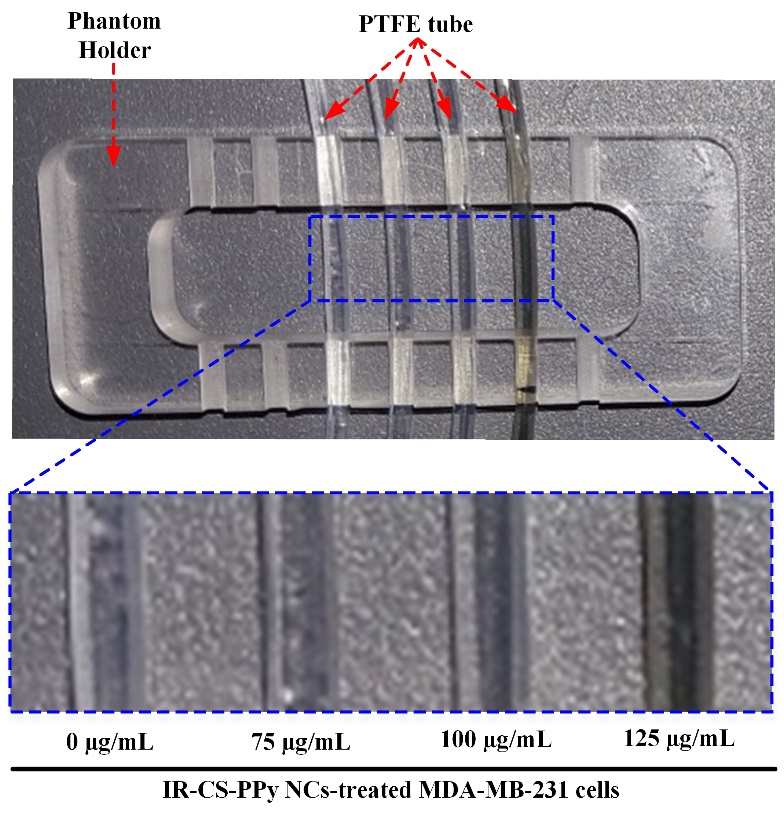


Figure S3. Diagram of the phantom holder, PTFE tube with control cells, and three different concentrations of IR-CS-PPy NCs-treated MDA-MB-231 cells.


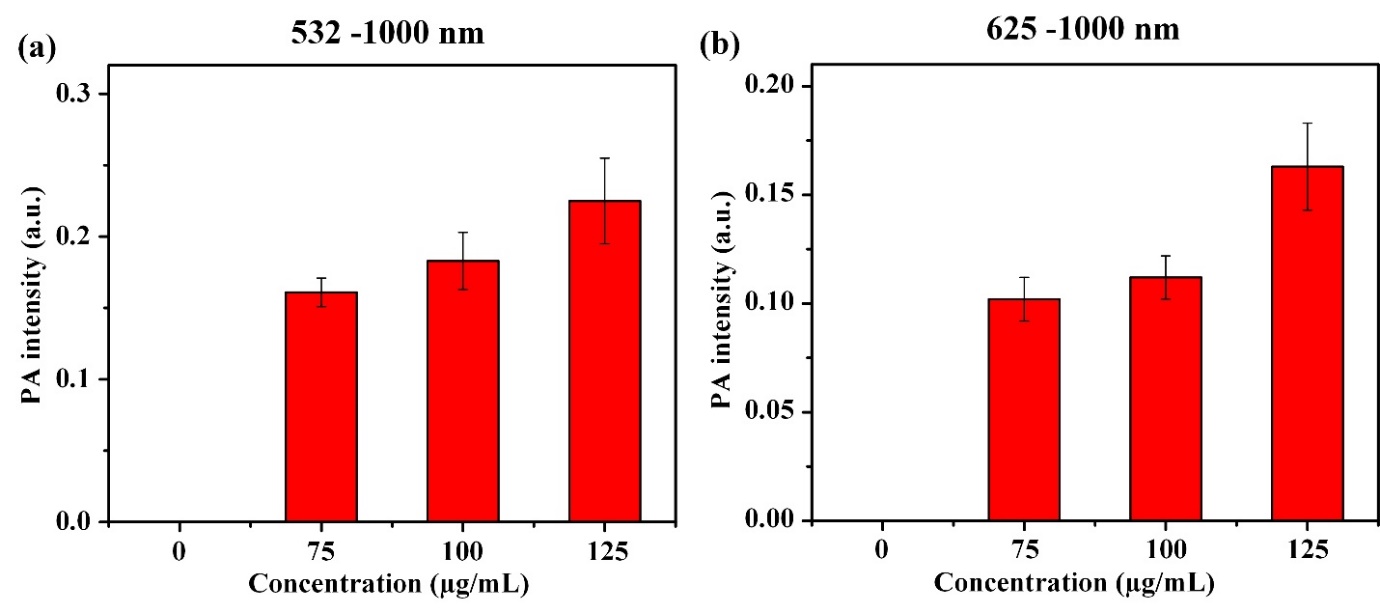


Figure S4. In vitro PA signals monitored in the MDA-MB-231 cells with various concentrations of IR-CS-PPy NCs using PAI system with (a) 532-1000 nm wavelength and (b) 625-1000 nm wavelength. Data were expressed as mean ± standard deviation (n = 3).


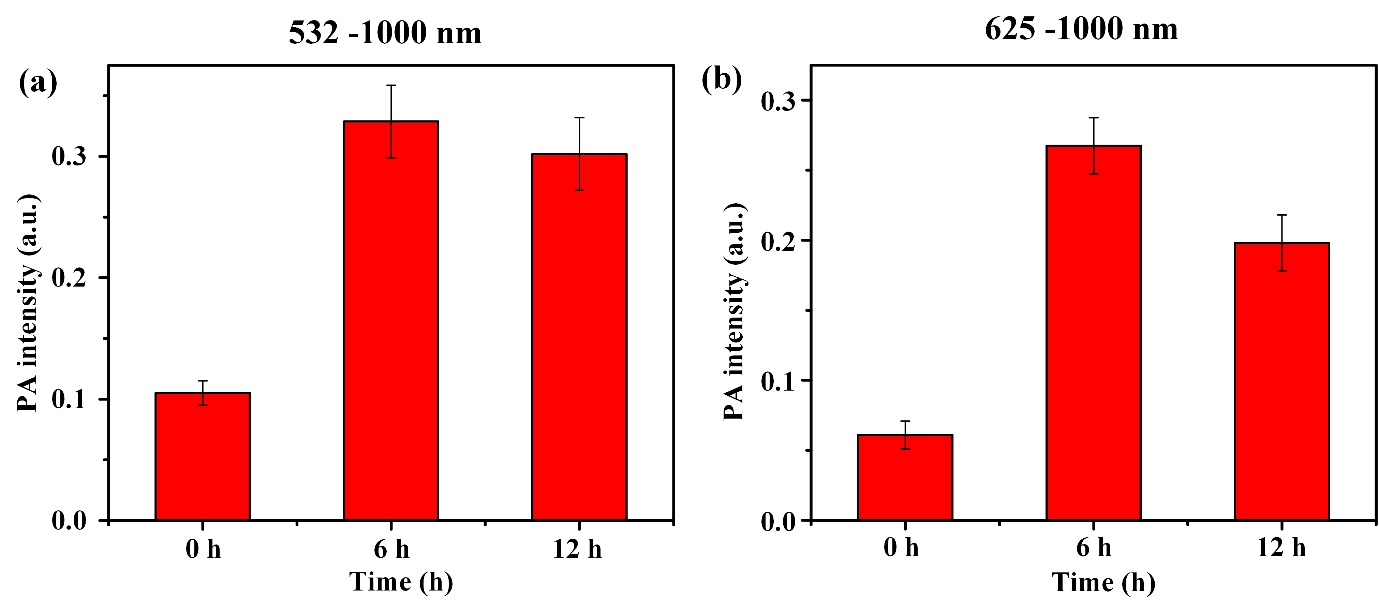


Figure S5. In vivo PA signals monitored in the MDA-MB-231 cells with different time point after injection of IR-CS-PPy NCs with (a) 532-1000 nm wavelength and (b) 625-1000 nm wavelength. Data were expressed as mean ± standard deviation (n = 3).


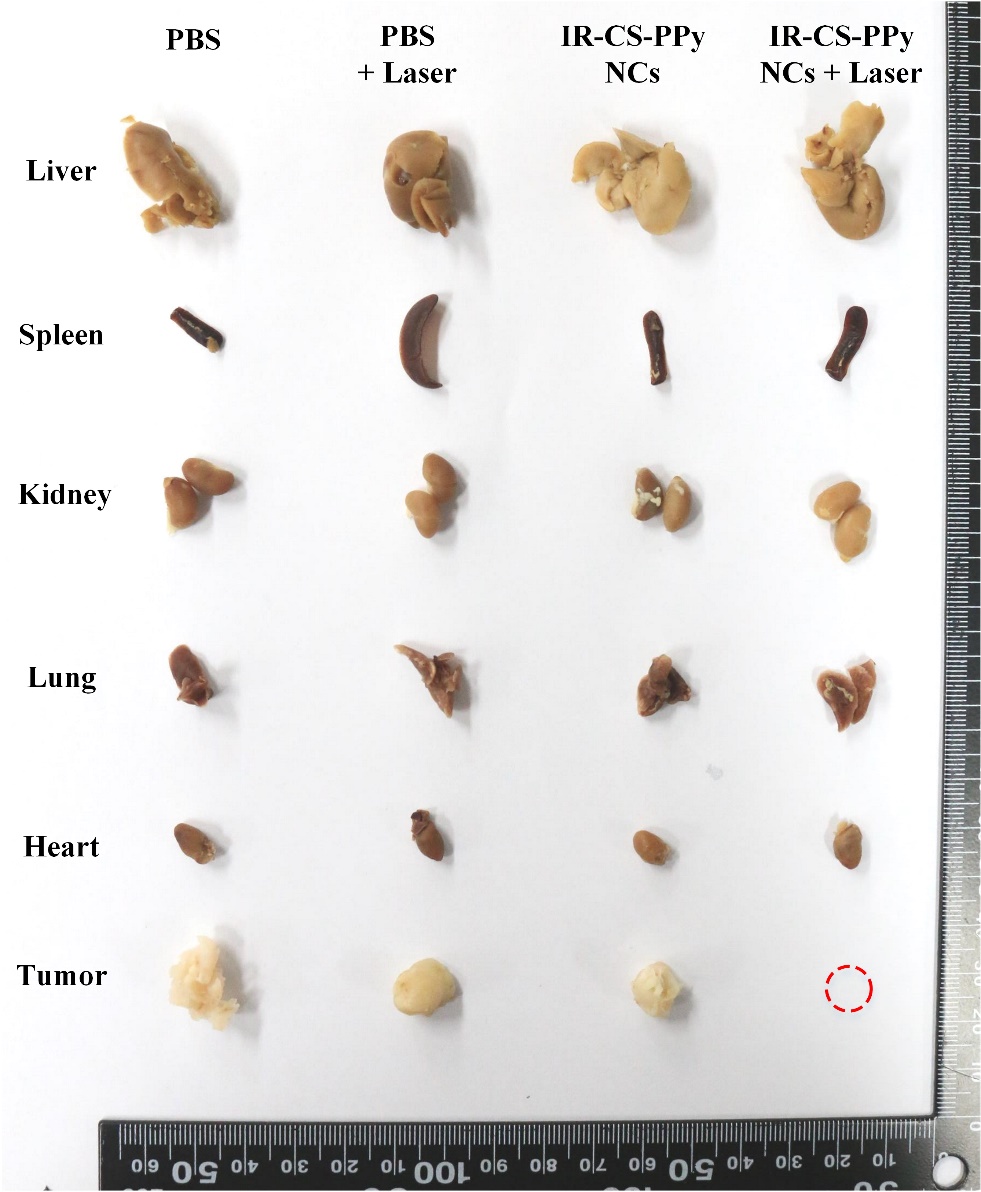


Figure S6. Images of five organ tissues (heart, liver, spleen, lung and kidney) and tumors from all groups after PTT for 20 days.


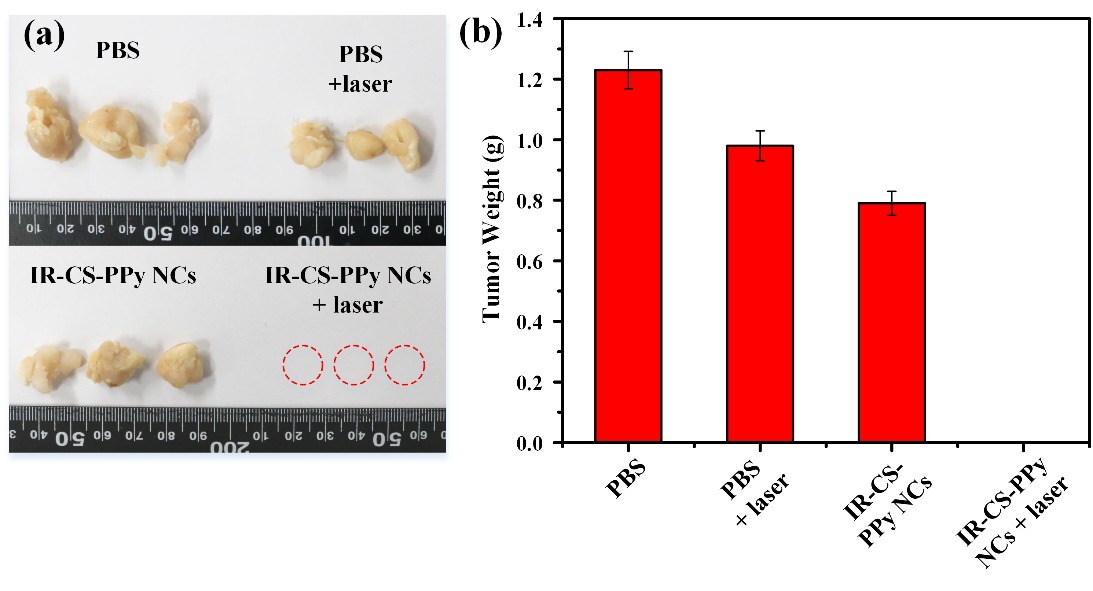


Figure S7. (a) The tumor images obtained from the sacrificed mice at the end of experiments. (b) The weight of tumor at different groups of nude mice after 20 days of only PBS, PBS + laser, IR-CS-PPy NCs and IR-CS-PPy NCs + laser irradiation, respectively. Data were expressed as mean ± standard deviation (n = 3).


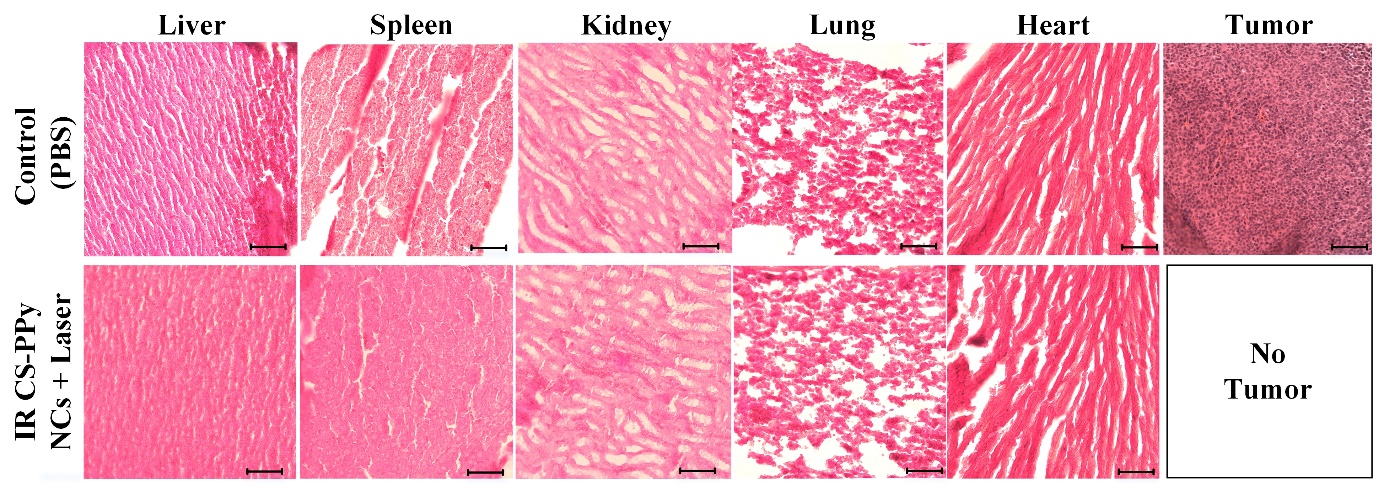


Fig. S8. H&E stained images of organs (heart, liver, lung, kidney, spleen and tumor) after treatments (Scale bar: 50 µm).

Calculation of the photothermal conversion efficiency

According to the previous method^1,2^, the photothermal conversion efficiency (*η)* of the IR-CS-PPy NCs was computed using Eq. (1):

| $\boldsymbol{=}\frac{\boldsymbol{hS}\left( \boldsymbol{T}_{\boldsymbol{Max}}\boldsymbol{-}\boldsymbol{T}_{\boldsymbol{Surr}} \right)\boldsymbol{-}\boldsymbol{Q}_{\boldsymbol{Dis}}}{\boldsymbol{I(1-}\boldsymbol{10}^{\boldsymbol{-}\boldsymbol{A}_{\boldsymbol{808}}}\boldsymbol{)}}$ | (1) |
| --- | --- |

in which *h* is the heat transfer coefficient and *S* is the surface area of the container. The maximum steady temperature (*T_Max_)* of the solution of the IR-CS-PPy NCs was 62.6 ^o^C and the ambient temperature (*T_Surr_*) was 20 ^o^C. So, the temperature change (*T_Max_*-*T_Surr_*) of the IR-CS-PPy NCs solution was 42.6 ^o^C. The laser power density was 2 W/cm^2^, the diameter of the container was 1 cm. So, the laser power (*I*) was 1.57 W. The absorbance of the IR-CS-PPy NCs at 808 nm (*A*_808_) is 1.65. *Q_Dis_* expresses heat dissipated from the light absorbed by the solvent and container.

In order to gain $\boldsymbol{hS}$, a dimensionless parameter $\boldsymbol{\theta}$ is introduced as followed:

| $\boldsymbol{\theta=}\frac{\boldsymbol{T-}\boldsymbol{T}_{\boldsymbol{Surr}}}{\boldsymbol{T}_{\boldsymbol{Max}}\boldsymbol{-}\boldsymbol{T}_{\boldsymbol{Surr}}}$ | (2) |
| --- | --- |

The value of $\boldsymbol{hS}$ can be obtained from Eq. (2), as follows:

| $\boldsymbol{t=-}{}_{\boldsymbol{s}}\mathbf{ln}\boldsymbol{(\theta)}$ | (3) |
| --- | --- |

According to figure 3b, ${}_{\boldsymbol{s}}$ was determined and calculated to be 156.48 s.

| $\boldsymbol{hS=}\frac{\boldsymbol{m}_{\boldsymbol{D}}\boldsymbol{C}_{\boldsymbol{D}}}{{}_{\boldsymbol{s}}}$ | (4) |
| --- | --- |

In addition, $\boldsymbol{m}$ is 0.3 g and $\boldsymbol{C}$ is 4.2 J/g·℃. Thus, according to Eq. (4), $\boldsymbol{hS}$ is calculated to be 8.052 mW/ ^o^C. *Q_Dis_* was measured independently to be 31.04 mW.

Thus, substituting corresponding values of each parameter to Eq. 1, the 980 nm laser heat conversion efficiency (*η*) of the IR-CS-PPy NCs can be calculated to be 20.29%. This result is similar to that of widely used gold nanorods (21%)^1,3^.


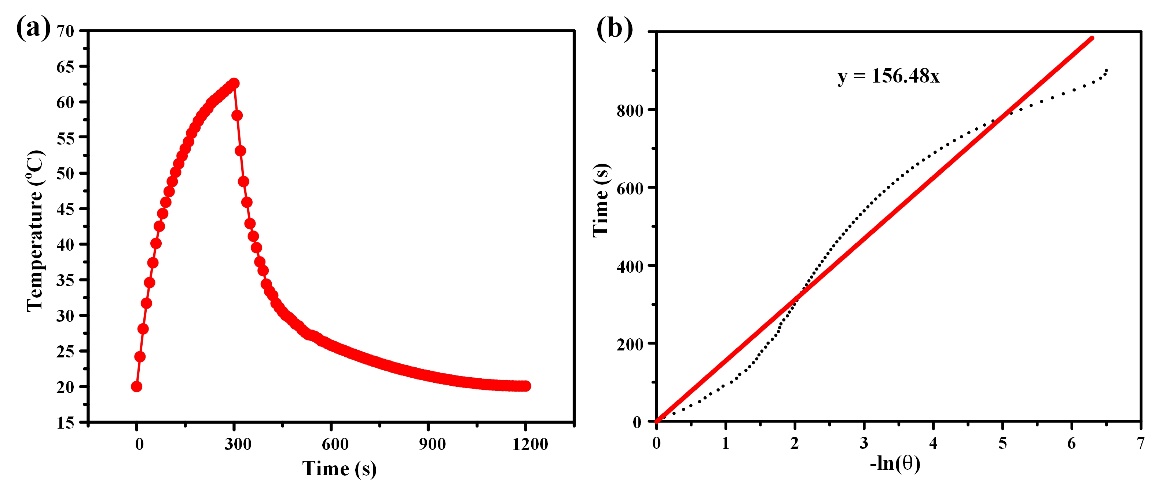


Figure S9: (a) Photothermal heating curve of the aqueous solution of IR-CS-PPy NCs (125 µg/mL) upon 808 nm laser irradiation (2 W/cm^2^), and then the laser was shut off. (b) Linear time data versus –ln (θ) obtained from the cooling period from Fig. S7a.

Characterization of the fluorescence system

We measured the spatial resolutions of our fluorence system based on a USAF resolution target. The smallest visible could be monitored by fluorescence system is the element 6 of group 1 (Fig. S1b). The resolution of the system can be calculated by the following equation:

| *Resolution* $\boldsymbol{=}\boldsymbol{2}^{\boldsymbol{Group+}\frac{\boldsymbol{Element - 1}}{\boldsymbol{6}}}$ | (5) |
| --- | --- |

The signal to noise ratio (SNR) of the fluorescence was calculated following the previously reported study^4^. The in vitro experiment result in Fig. 6a was used to calculate the fluorescence SNR. We need to measure the well with no fluorophore concentration as a mean background value ($\boldsymbol{S}_{\boldsymbol{B}}$), mean fluorescence intensity ($\boldsymbol{S}_{\boldsymbol{I}}$), and standard deviation of the background well ($\boldsymbol{\sigma(}\mathbf{S}_{\mathbf{I}}\mathbf{)}$). The SNR was then calculated using the following relation:

| $\boldsymbol{SNR=}\frac{\boldsymbol{S}_{\boldsymbol{I}}\boldsymbol{-}\boldsymbol{S}_{\boldsymbol{B}}}{\boldsymbol{\sigma(}\boldsymbol{S}_{\boldsymbol{B}}\boldsymbol{)}}$ | (6) |
| --- | --- |

Characterization of the photoacoustic imaging system

To measure the spatial resolution of our photoacoustic imaging (PAI) system by scanning a carbon fiber with a diameter of 6 $\boldsymbol{\mu}$m. By using a line spread function (LSF), we estimate the lateral resolution as 7$\boldsymbol{\mu}$m and the axial resolution as 75$\boldsymbol{\mu}$m (Fig. S2a and S2b).

The penetration depth of our PAI system was measured by scanning the oblique black tape into breast chicken tissue (Fig. S2c).

The in vitro experiment result in Fig. 7a (532-1000 nm) was used to calculate the PA SNR. The PA intensity value of control tube was considered as the mean background value ($\boldsymbol{S}_{\boldsymbol{B}}$). We used the same method as when we calculate the fluorescence SNR for measuring the PA SNR (Eq. 6).

Calculation of nanoparticles concentration from UV-vis absorption spectra data

| $\boldsymbol{\alpha=}\frac{\boldsymbol{(2.303\times A)}}{\boldsymbol{l}}$ | (7) |
| --- | --- |

where, α is absorption, $\boldsymbol{A}$ :absorbance, $\boldsymbol{l}$: thickness (usually 1 cm)

| $\boldsymbol{k}\left( \boldsymbol{\varepsilon} \right)\boldsymbol{=}\frac{\boldsymbol{\alpha\lambda}}{\boldsymbol{4}\boldsymbol{\pi}}$ | (8) |
| --- | --- |

where, $\boldsymbol{k}\left( \boldsymbol{\varepsilon} \right)$: extinction coefficient, $\boldsymbol{\alpha}$∶ absorption coefficient, λ: wavelength

By Beer-Lambert Law $\boldsymbol{A= \varepsilon\times l\times c}$, where $\boldsymbol{\varepsilon}$: molar absorptivity (extinction coefficient), $\boldsymbol{l}$: thickness (usually 1 cm), $\boldsymbol{c}$: molar concentration (M),

| $\boldsymbol{c=}\frac{\boldsymbol{A}}{\boldsymbol{\varepsilon\times l}}\boldsymbol{=}\frac{\boldsymbol{A}}{\frac{\boldsymbol{\alpha\lambda}}{\boldsymbol{4}\boldsymbol{\pi}}\boldsymbol{\times l}}\boldsymbol{=}\frac{\boldsymbol{A}}{\frac{\frac{\boldsymbol{2.303\times A}}{\boldsymbol{l}}\boldsymbol{\times\lambda}}{\boldsymbol{4}\boldsymbol{\pi}}\boldsymbol{\times l}}$ | (9) |
| --- | --- |

After calculating $\boldsymbol{c}$ (molar concentration (M)), we could plot Absorbance vs concentration (M).

# **References**

1. Liu, X. *et al.* Facile synthesis of biocompatible cysteine-coated CuS nanoparticles with high photothermal conversion efficiency for cancer therapy. *Dalt. Trans.* 43, 11709–11715 (2014).

2. Tian, Q. *et al.* Hydrophilic Cu 9S 5 nanocrystals: A photothermal agent with a 25.7% heat conversion efficiency for photothermal ablation of cancer cells in vivo. *ACS Nano* 5, 9761–9771 (2011).

3. Wang, B. *et al.* Rose-bengal-conjugated gold nanorods for invivo photodynamic and photothermal oral cancer therapies. *Biomaterials* 35, 1954–1966 (2014).

4. Kanniyappan, U. *et al.* Performance test methods for near-infrared fluorescence imaging. *Med. Phys.* 47, 3389–3401 (2020).
